# Lipidomic analysis of brain and hippocampus from mice fed with high-fat diet and treated with fecal microbiota transplantation

**DOI:** 10.1186/s12986-023-00730-7

**Published:** 2023-02-15

**Authors:** Jinchen Li, Hongying Huang, Rong Fan, Yinan Hua, Weiwei Ma

**Affiliations:** Beijing Key Laboratory of Environmental Toxicology, School of Public Health, Capital Medical University, Beijing, China

**Keywords:** Dietary fatty acid, Lipidomics, High-fat diet, FMT, Brain lipid

## Abstract

**Background:**

Dietary fat intake affects brain composition and function. Different types of dietary fatty acids alter species and abundance of brain lipids in mice. The aim of this study is to explore whether the changes are effective through gut microbiota.

**Methods:**

In our study, 8-week-old male C57BL/6 mice were randomly divided into 7 groups and fed with high-fat diet (HFD) with different fatty acid compositions, control (CON) group, long-chain saturated fatty acid (LCSFA) group, medium-chain saturated fatty acid (MCSFA) group, n-3 polyunsaturated fatty acid (n-3 PUFA) group, n-6 polyunsaturated fatty acid (n-6 PUFA) group, monounsaturated fatty acid (MUFA) group and trans fatty acid (TFA) group. Then, the fecal microbiota transplant (FMT) was performed in other pseudo germ-free mice after antibiotic treatment. The experimental groups were orally perfused with gut microbiota that induced by HFD with different types of dietary fatty acids. The mice were fed with regular fodder before and after FMT. High-performance liquid chromatography-mass spectrometry (LC-MS) was used to analysis the composition of fatty acids in the brain of HFD-fed mice and hippocampus of mice treated with FMT which was collected from HFD-fed mice.

**Results:**

The content of acyl-carnitines (AcCa) increased and lysophosphatidylgylcerol (LPG) decreased in all kinds of HFD groups. phosphatidic acids (PA), phosphatidylethanolamine (PE) and sphingomyelin (SM) contents were significantly increased in the n-6 PUFA-fed HFD group. The HFD elevated the saturation of brain fatty acyl (FA). Lysophosphatidylcholine (LPC), lysodi-methylphosphatidylethanolamine (LdMePE), monolysocardiolipin (MLCL), dihexosylceramides (Hex2Cer), and wax ester (WE) significantly increased after LCSFA-fed FMT. MLCL reduced and cardiolipin (CL) raised significantly after n-3 PUFA-fed FMT.

**Conclusions:**

The study revealed, HFD and FMT in mice had certain effects on the content and composition of fatty acids in the brain, especially on glycerol phospholipid (GP). The change of AcCa content in FA was a good indicator of dietary fatty acid intake. By altering the fecal microbiota, dietary fatty acids might affect brain lipids.

**Supplementary Information:**

The online version contains supplementary material available at 10.1186/s12986-023-00730-7.

## Introduction

Dietary fatty acids regulate brain lipid composition [1]. Brain lipids in turn exert their effects on the brain physiological function through participating in biochemical reactions. It has been reported that high-fat diet (HFD) could reduce the contents of phosphatidylserine (PS) and phosphatidylethanolamine (PE) significantly [2]. Meanwhile, docosahexaenoic acid (DHA)-containing phosphatidylcholine (PC) and DHA-containing PS have restored lipid homeostasis in dementia mice [3]. Ethanolamine plasmalogen is the main component of plasmalogen in the brain [3]. Sun *et al.* [4] have found that DHA-enriched diet could increase species of n-3 polyunsaturated fatty acid (n-3 PUFA) and decrease n-6 polyunsaturated fatty acid (n-6 PUFA) in all major phospholipid classes stored in the hippocampus, including PC, diacyl-phosphatidylethanolamine (dPE), alkenylacyl-phosphatidylethanolamine or phosphatidylethanolamine plasmalogen (PE-pl), phosphatidylinositol (PI) and PS. Evidence showed that eicosapentaenoic acid (EPA)-enriched PC and PE could restore lipid homeostasis in dementia mice, dietary EPA-PC and EPA-PE could increase the amount of choline plasmalogen, lysophosphatidylethanolamine (LPE), arachidonic acid (AA)-containing PE and PS as well as decrease docosapentaenoic acid (DPA)-containing PS in the cerebral cortex of senescence accelerated mouse prone-8 mice fed with HFD [5]. It has been reported that HFD elevated most kinds of lipids such as diacylglycerol lysophosphatidylserine and ceramide (Cer) in brain regions while PS species were decreased, the changes played a role in insulin resistance and oxidative stress [6].

In addition, the obese mice fed with HFD exhibited brain hypofunction and depression phenotype in behavioral experiments via the molecular signaling cascades, related enzymes and genes in the hypothalamus [7]. Results from our previous study based on 8-week-old male C57BL/6 mice indicated that HFD enriched in n-3 PUFA might impact cognition favorably, by contrast, HFD riched in long-chain saturated fatty acid (LCSFA), medium-chain saturated fatty acid (MCSFA), n-6 PUFA, monounsaturated fatty acid (MUFA) or trans fatty acid (TFA) exerted adverse effects on cognitive performance [8].

Accordingly, lipids and their metabolism are essential components for normal structure and function in brain. Disorders of brain lipids have been strongly implicated in the neurodegenerative disorders. Sulfatide (ST), a kind of sulfated galactocerebrosides synthesized by the oligodendrocytes in central nervous system, was depleted in early Alzheimer's disease (AD), while Cer as degradation products of ST was elevated leading to neuronal dysfunction and neurodegeneration [9]. PS is the main category of acidic phospholipid class which stimulates neuronal survival and growth and participates in signal transmission [10]. Furthermore, abnormal PS asymmetry was found in synaptic membrane in patients with mild cognitive impairment and AD [11].

Diets rich in different dietary fatty acids exhibit impacts on the diversity of gut microbiota. Garcia *et al.* [12] found that the genus *Dorea* and *Lactobacillus* were overrepresented in diets with a high PUFA/saturated fatty acid (SFA) ratio in adults without pathology. A study in nonhuman primates showed that participants consuming a high MUFA diet had a more diverse microbiome than participants on a high-SFA diet, with increases in *Oscillospira*, *Fecalibacterium*, *Clostridium*, and *Lactobacillus* and decreases in *Coprococcus* and *Ruminococcus* found in the gut [13]. Mice fed with the SFA-rich diet showed a greater decrease in *Bacteroidetes* proportion than did either the n-3 PUFA or the n-6 PUFA-rich diet treatment, and significant decreases of *Porphyromonadaceae* and *Lachnospiraceae* were observed at the family level in n-6 PUFA and the SFA supplements, respectively [14].

Thus, the change in brain lipidomics might be an important factor related to energy metabolism [15], mitochondrial function [16], inflammatory response [17], and cognitive function [18]. Therefore, it is sufficient to understand the effect of dietary fat on brain lipidomics to prevent and manage nervous system disease as well as later life consequences. In the present study, we investigated the effects of different dietary fatty acids on brain lipidomics followed by the intervention of dietary fatty acids and fecal microbiota transplant (FMT) in mice. Our study provides dietary fatty acids and altered gut microbiota as possible targets for further brain dysfunction research exploration.

## Material and methods

### Animals and diets

#### Orally feed intervention

Healthy male specific pathogen free (SPF) grade 8-week-old C57BL/6 mice were used in the experiment [8]. A total of 70 mice were under permit number SCXK (Beijing) 2016-0006. The animal experiments were approved by the Animal Ethics Committee of Capital Medical University (No. AEEI-2018-061). During the experiment, the mice were housed in the SPF environment under conditions of controlled temperature (20–23 °C), natural lighting, and humidity (50–55%) with free access to food and water ad libitum. The mice were randomly divided into 7 groups according to their body mass, with 10 mice in each group. The fat energy supply ratio of the basic feed was 10%, and the carbohydrate energy supply ratio was 70% for control (CON) group. The obesity mouse model was established by using 45% fat energy supply ratio and 35% carbohydrate energy supply ratio in experimental group. The detailed feed nutritional composition could be found in our published research [19]. The specific HFD-fed mice groups were as follows: (1) CON group: fed with basic diet for 19 weeks; (2) LCSFA group: fed with lard HFD for 19 weeks; (3) MCSFA group: fed with coconut oil HFD for 19 weeks; (4) n-3 PUFA group: fed with flaxseed oil HFD for 19 weeks; (5) n-6 PUFA group: fed with soybean oil HFD for 19 weeks; (6) MUFA group: fed with olive oil HFD for 19 weeks; (7) TFA group: fed with 8% hydrogenated soybean oil HFD for 19 weeks. All feeds were prepared and provided by Beijing Keao Xieli Feed Co., Ltd.

After 19 weeks of intervention, mice were fasted for 12 h and sacrificed. The brains were quickly separated then frozen and stored at −80 °C.

#### FMT intervention

Mice were reared in the SPF Laboratory Animal Center under permit number SCXK (Beijing) 2016-0011. In our experiment, the mice were disposed of strictly according to the Ethical Committees on Animal Research (Animal Experimental Ethical Inspection Form Number: AEEI-2018-131). The SPF grade 3-week-old C57BL/6J mice (n = 90) were used for this study. The mice were fed with autoclaved feed (D12450H with a fat content of 10%) and water. After 2 weeks of adaptive feeding, mice were randomly separated into 9 groups based on their initial body weight with 10 mice in each group, mice with FMT were grouped as follows: (1) + CON-0 group: perfused with only normal saline without antibiotic treatment; (2) + CON-1 group: perfused with normal saline after antibiotic treatment; (3) + CON group: FMT from basic feed mice after antibiotic treatment; (4) + LCSFA group: FMT from LCSFA feed mice after antibiotic treatment; (5) + MCSFA group: FMT from MCSFA feed mice after antibiotic treatment; (6) + n-3 PUFA group: FMT from n-3 PUFA feed mice after antibiotic treatment; (7) + n-6 PUFA group: FMT from n-6 PUFA feed mice after antibiotic treatment; (8) + MUFA group: FMT from MUFA feed mice after antibiotic treatment; (9) + TFA group: FMT from TFA feed mice after antibiotic treatment. Antibiotic treatment: mice were treated with broad spectrum antibiotics (ampicillin 1 g/L, neomycin sulfate 1 g/L, metronidazole 1 g/L, and vancomycin 0.5 g/L) in their drinking water, which was replaced every two days for 4 weeks [20].

Based on the prior experiment, fresh fecal pellets were collected for fecal bacteria preservation solution [21]. The percentage of preservation solution was calculated by the weight as follows: preservation solution was consisted of sodium chloride (0.9%), Vitamin C (0.5% – 2.0%), glycerol (30%) and sterile purified water. Fresh fecal samples were resuspended in a preservation solution (50 g/100 ml) and then stored at −80 °C [21]. Aliquots were thawed on ice and performed with a total volume of 250 ml sterile normal saline. After centrifugation at 4 °C, 800r/min for 3 min, the supernatant was taken as lavage fluid [22]. Each mouse received 200 μl by oral gavage once a week for 10 weeks. We used different gavage needles between groups and cleaned gavage needles with 70% ethanol within a group [23]. All equipment was autoclaved after experiments every day.

**Fig. 1:**
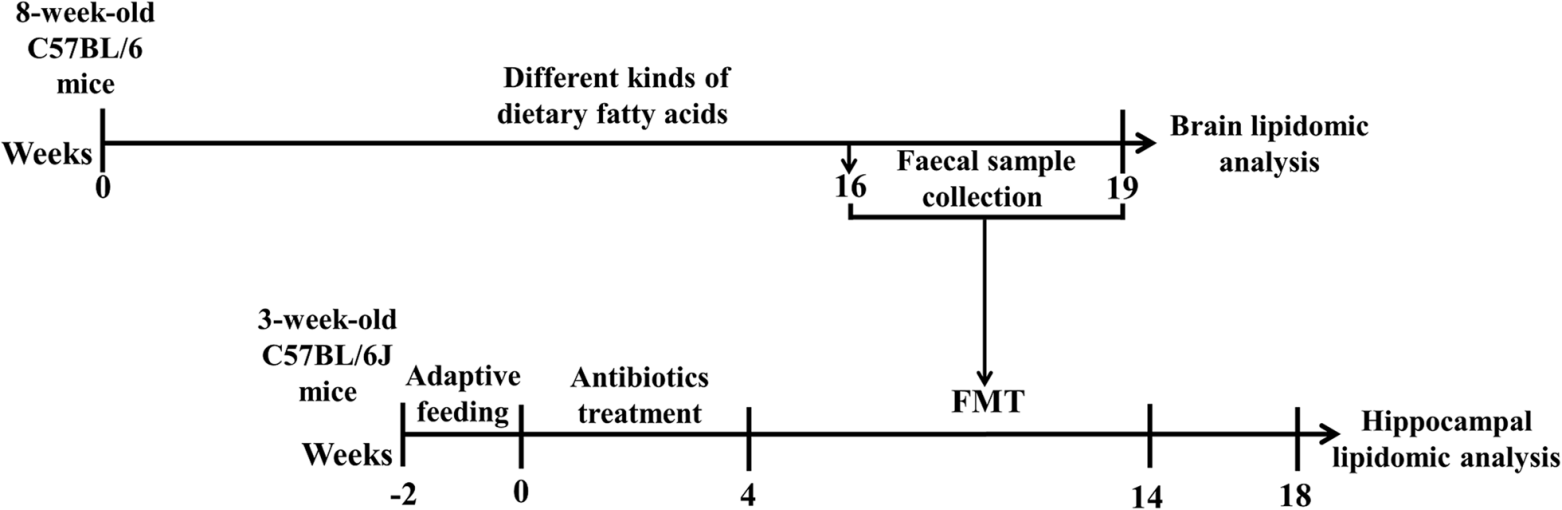
Flow diagram of the study

All mice were fasted for 12 h and sacrificed, the brain tissues were isolated, then the hippocampal tissues were extracted. Samples were stored at −80 °C for subsequent experiment. Flow chart of the study was presented in Fig. 1. The data of body weight and body fat mass of mice was detailed in our published research [8].

### Lipidomic analysis

#### Sample processing

Total lipids were extracted from brain and hippocampus by using a 2:5 mixture of methanol and pure water. Extracts were dried under vacuum and then redissolved in acetonitrile/isopropanol (1:1). 80 μl of supernatant was injected into the sample injection bottle for UPLC-MS/MS analysis.

#### High-performance liquid chromatography-mass spectrometry (LC-MS) of brain tissue lipid extracts

After sample processing, LC-MS was performed on a Thermo UHPLC-Q Exactive system. Chromatographic separation was performed at 25 °C on a BEH C18 -column (100 mm × 2.1 mm i.d., 1.7 μm; Waters, Milford, USA). Solvent A was 10 mM CH3COONH4 in ACN/H2O (1/1) (0.1% (v/v) formic acid) and Solvent B was 2 mM CH3COONH4 in ACN/IPA/H2O (10/88/2) (0.02% (v/v) formic acid). Injection volume was 2.0 μl, flow rate was 0.4 ml/min, and column temperature was set at 40 °C.

The Thermo UHPLC-Q Exactive Mass Spectrometer equipped with an electrospray ionization positive and negative ion modes was used for mass spectrometer detection.

#### Quality controlling

To analyze the stability of the treatment method, The quality control samples were prepared by mixing the experimental sample extracts in equal amounts to ensure the reliability of the experimental results. Samples treatment and data preprocessing were performed by Shanghai Majorbio Bio-pharm Technology Co., Ltd.

### Statistical analysis

The raw data were imported into the LipidSearch software for baseline filtering, peak detection, integration, time correction and peak alignment. The obtained data matrix of retention time, mass-to-charge ratio and peak area were used for data preprocessing: (1) The variables with non-zero expression in at least 80% of the samples were retained. (2) Missing values were filled up in the raw data. (3) Peaks were normalized and variables having relative standard deviation higher than 30% in quality control samples were excluded for further analysis. (4) All data were log10 transformed before quantitative assays.

Statistical analyses were performed using R software (Version 1.6.2). Log-transformation was applied to approximate log-normality of the data and one-way analysis of variance (ANOVA). Kruskal-Wallis test was performed if the normality test is not satisfied. Results were presented as mean and standard deviation (SD) in tables and figures. A two-sided *p* < 0.05 was considered statistically significant.

## Results

### The variations in the abundance of glycerol phospholipid (GP) in brain of HFD-fed mice

**Fig. 2:**
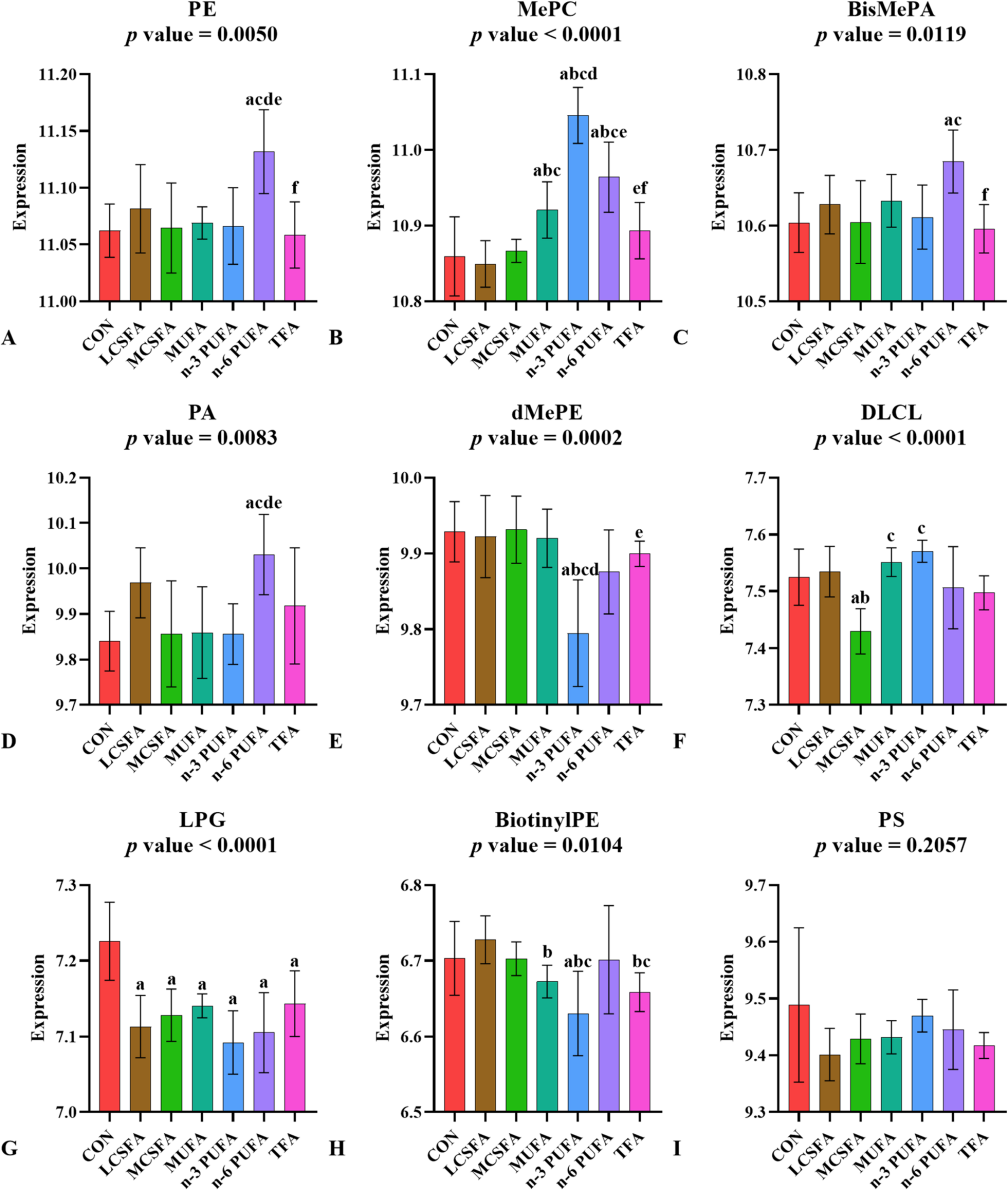
Relative abundance of GP in brain of mice fed with HFD. **A**–**I:** GP as a main class can be subdivided into classes: PE, MePC, BisMePA, PA, dMePE, DLCL, LPG, BiotinylPE and PS (Mean ± SD, n = 6). **a:** *p* < 0.05, compared to CON group; **b:** *p* < 0.05, compared to LCSFA group; **c:** *p* < 0.05, compared to MCSFA group; **d:** *p* < 0.05, compared to MUFA group; **e:** *p* < 0.05, compared to n-3 PUFA group; **f:** *p* < 0.05, compared to n-6 PUFA group. Mean ± SD, n = 6

GP, such as PE and PI, is one of the most abundant lipids in the cerebral cortex. In Fig. 2, results showed the content and types changes of GP in the mice brain after a 19-week fatty acids dietary intervention. High intake of n-6 PUFA elevated the content of PE in the mice brain (*p* < 0.05) (Fig. 2A).

The expression of PS indicated almost no difference between experimental groups and CON group (*p* > 0.05) (Fig. 2I), in the meanwhile, compared with CON group, PE, methylphosphocholine (MePC), BisMePA, phosphatidic acids (PA) showed a highlight rise in n-6 PUFA group (*p* < 0.05) (Fig. 2A–D). However, dimethylphosphatidylethanolamine (dMePE) and biotinyl-phosphatidylethanolamine (BiotinylPE) were significantly reduced in n-3 PUFA group compared to CON group (*p* < 0.05) (Fig. 2E, H). MePC was sensitive to the dietary fatty acid composition and capable of reflecting the type changes: the MePC content was significantly increased in MUFA, n-3 PUFA and n-6 PUFA groups versus the CON group (*p* < 0.05) (Fig. 2B). The increase of dietary MCSFA intake was reflected by the reduction of dilyso-cardiolipin (DLCL) content in the brain (*p* < 0.05) (Fig. 2F).

Although at the main class level, PE increased significantly only in n-6 PUFA group (*p* < 0.05) (Fig. 2A). But under the condition of high LCSFA intake, the specific metabolites in PE: PE (16:1/22:5), PE (16:0e/22:6), PE (20:4e/18:2), PE (18:2p/20:4), PE (16:1/22:6), PE (20:5/20:5), PE (18:2p/22:6) and PE (18:3/22:6) were still significantly higher than those in the normal diet CON group (*p* < 0.05) (see Additional file 1: Table S1). And under the condition of high MCSFA intake, the specific metabolites in PE: PE (16:0p/16:1), PE (14:0/20:4), PE (16:1p/20:4) and PE (14:0/22:6) were still significantly higher than those in the normal diet CON group (*p* < 0.05) (see Additional file 1: Table S1).

The content of lysophosphatidylgylcerol (LPG) decreased in all LCSFA, MCSFA, MUFA, n-3 PUFA, n-6 PUFA and TFA groups compared with CON group (*p* < 0.05) (Fig. 2G). Compared to the types of dietary fatty acids, the high total content of dietary fatty acids had more effect on the content of LPG in mice brains.

### The variations in the abundance of fatty acyl (FA) in brain of HFD-fed mice

**Fig. 3:**
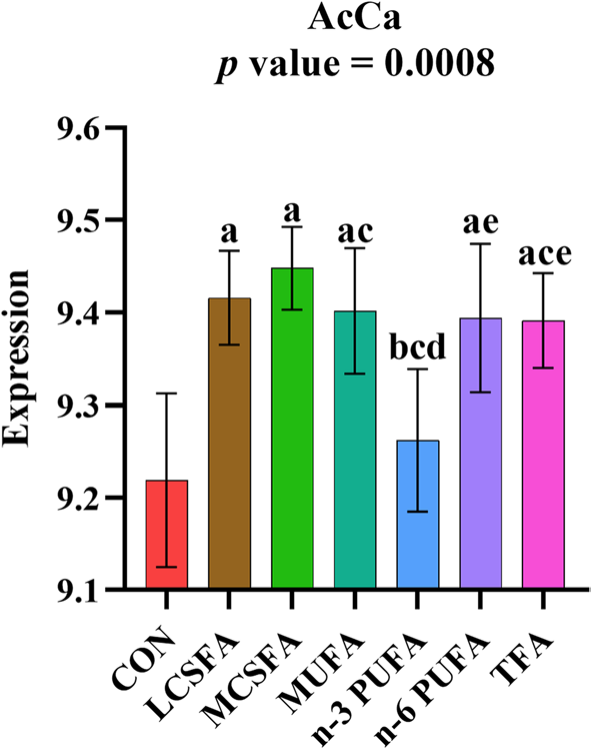
Relative abundance of the AcCa class in FA main class in brain of mice fed with HFD. **a:** *p* < 0.05, compared to CON group; **b:** *p* < 0.05, compared to LCSFA group; **c:** *p* < 0.05, compared to MCSFA group; **d:** *p* < 0.05, compared to MUFA group; **e:** *p* < 0.05, compared to n-3 PUFA group; **f:** *p* < 0.05, compared to n-6 PUFA group. Mean ± SD, n = 6

The results revealed that high-content of dietary fatty acids had a large effect on the content of acyl-carnitines (AcCa) in the brain. Compared with the CON group, the increase of AcCa expression was indicated in all experimental groups, and the increases were statistically significant (*p* < 0.05) except in n-3 PUFA group (Fig. 3).

Data were displayed in Table 1. Compared with CON group, the main metabolites in AcCa class increased obviously (*p* < 0.05), including AcCa (12:0), AcCa (13:0), AcCa (14:0), AcCa (14:1), AcCa (15:0), AcCa (16:0), AcCa (16:1), AcCa (17:1), AcCa (18:0), AcCa (18:1), AcCa (18:2), AcCa (18:3), AcCa (19:1), AcCa (20:1), AcCa (20:3), AcCa (20:4), AcCa (22:4) and AcCa (22:6) in most experimental groups. In the whole class of AcCa, the increase of molecular contents more than 12 carbon accounted for the main position. Compared with high SFA and high MUFA diet, the effect of high n-3 PUFA intake on the contents of AcCa (20:1), AcCa (20:4) and AcCa (22:1) in hippocampus was completely consistent with the decrease of total AcCa (*p* < 0.05). The increased content of AcCa implied the accumulation of medium and long chain fatty acids, which might be attributed to the HFD.

**Table 1:** AcCa expression in the brain of mice fed with different dietary fatty acids (Mean ± SD, n = 6)

Expression	CON	LCSFA	MCSFA	MUFA	n-3 PUFA	n-6 PUFA	TFA	*p* value
AcCa (12:0)	6.706 ± 0.142	7.021 ± 0.093^a^	7.943 ± 0.085^ab^	7.031 ± 0.151^ac^	6.967 ± 0.116^ac^	7.075 ± 0.098^ac^	7.035 ± 0.137^ac^	< 0.001***
AcCa (13:0)	5.957 ± 0.108	6.226 ± 0.083^a^	6.289 ± 0.057^a^	6.173 ± 0.090^a^	6.094 ± 0.111^c^	6.228 ± 0.068^a^	6.230 ± 0.087^a^	< 0.001***
AcCa (14:0)	8.087 ± 0.151	8.302 ± 0.073^a^	8.499 ± 0.052^ab^	8.293 ± 0.088^ac^	8.247 ± 0.091^c^	8.328 ± 0.071^ac^	8.350 ± 0.073^a^	< 0.001***
AcCa (14:1)	6.857 ± 0.159	7.131 ± 0.064^a^	7.304 ± 0.066^a^	7.192 ± 0.121^a^	6.987 ± 0.139^ cd^	7.190 ± 0.071^ae^	7.277 ± 0.069^ae^	< 0.001***
AcCa (15:0)	6.698 ± 0.134	6.926 ± 0.077^a^	6.947 ± 0.119^a^	6.849 ± 0.212	6.819 ± 0.095	6.937 ± 0.104^a^	6.944 ± 0.078^ae^	0.028*
AcCa (16:0)	8.720 ± 0.108	8.904 ± 0.051^a^	8.893 ± 0.045^a^	8.871 ± 0.079^a^	8.789 ± 0.065	8.892 ± 0.075^a^	8.875 ± 0.047^a^	< 0.001***
AcCa (16:1)	7.744 ± 0.168	7.981 ± 0.076^a^	8.042 ± 0.051^a^	7.967 ± 0.090^a^	7.838 ± 0.102^c^	7.941 ± 0.092^a^	8.050 ± 0.063^ae^	< 0.001***
AcCa (17:1)	6.470 ± 0.154	6.766 ± 0.071^a^	6.680 ± 0.056^a^	6.694 ± 0.072^a^	6.579 ± 0.101^b^	6.737 ± 0.101^a^	6.757 ± 0.085^ae^	< 0.001***
AcCa (18:0)	8.298 ± 0.093	8.463 ± 0.032^a^	8.453 ± 0.045^a^	8.407 ± 0.054	8.303 ± 0.046^bc^	8.458 ± 0.104^ae^	8.348 ± 0.054	< 0.001***
AcCa (18:1)	8.572 ± 0.120	8.822 ± 0.054^a^	8.784 ± 0.087^a^	8.805 ± 0.088^a^	8.636 ± 0.101^bd^	8.742 ± 0.095^a^	8.802 ± 0.061^ae^	< 0.001***
AcCa (18:2)	6.709 ± 0.118	7.200 ± 0.091^a^	6.907 ± 0.122^b^	6.968 ± 0.099^ab^	7.209 ± 0.143^acd^	7.588 ± 0.137^abcde^	7.056 ± 0.198^af^	< 0.001***
AcCa (18:3)	5.263 ± 0.734	6.116 ± 0.086^a^	5.790 ± 0.341	6.026 ± 0.369	7.133 ± 0.201^abcd^	6.685 ± 0.124^abcde^	6.171 ± 0.244^acef^	< 0.001***
AcCa (19:1)	6.368 ± 0.110	6.558 ± 0.101^a^	6.517 ± 0.091	6.558 ± 0.097^a^	6.394 ± 0.099	6.558 ± 0.097^a^	6.507 ± 0.082	0.003**
AcCa (20:0)	7.117 ± 0.173	7.262 ± 0.108	7.169 ± 0.172	7.139 ± 0.054^b^	6.995 ± 0.071^bd^	7.187 ± 0.073^e^	7.073 ± 0.097^bf^	0.010**
AcCa (20:1)	7.564 ± 0.121	7.769 ± 0.065^a^	7.744 ± 0.130^a^	7.745 ± 0.076^a^	7.460 ± 0.100^bcd^	7.684 ± 0.072^e^	7.677 ± 0.055^e^	< 0.001***
AcCa (20:3)	6.356 ± 0.168	6.631 ± 0.054^a^	6.623 ± 0.034^a^	6.710 ± 0.054^abc^	6.822 ± 0.124^abc^	6.766 ± 0.123^ac^	6.564 ± 0.046^acdef^	< 0.001***
AcCa (20:4)	8.367 ± 0.082	8.526 ± 0.067^a^	8.608 ± 0.056^a^	8.587 ± 0.043^a^	8.260 ± 0.105^bcd^	8.517 ± 0.095^ae^	8.520 ± 0.075^ae^	< 0.001***
AcCa (21:0)	6.215 ± 0.117	6.325 ± 0.143	6.378 ± 0.138	6.343 ± 0.103	6.137 ± 0.189	6.322 ± 0.224	6.241 ± 0.147	0.174
AcCa (22:0)	6.284 ± 0.270	6.515 ± 0.192	6.540 ± 0.237	6.495 ± 0.108	6.254 ± 0.250	6.463 ± 0.251	6.232 ± 0.182	0.059
AcCa (22:1)	7.077 ± 0.106	7.233 ± 0.153	7.202 ± 0.199	7.250 ± 0.120	6.860 ± 0.214^bcd^	7.104 ± 0.170	7.062 ± 0.140	0.003**
AcCa (22:4)	6.906 ± 0.264	7.143 ± 0.051	7.178 ± 0.076^a^	7.066 ± 0.144	6.574 ± 0.156^abcd^	7.040 ± 0.267^e^	7.033 ± 0.208^e^	0.005**
AcCa (22:6)	7.308 ± 0.138	7.423 ± 0.108	7.488 ± 0.082^a^	7.519 ± 0.063^a^	7.248 ± 0.134^ cd^	7.413 ± 0.124	7.422 ± 0.167	0.010**
AcCa (24:1)	6.640 ± 0.190	6.794 ± 0.176	6.855 ± 0.236	6.847 ± 0.173	6.540 ± 0.300	6.710 ± 0.314	6.590 ± 0.168	0.118

*, **, ***denotes a statistically significant difference (*p* < 0.05, 0.01, 0.001) in all groups

^a^*p* < 0.05, compared to CON group

^b^*p* < 0.05, compared to LCSFA group

^c^*p* < 0.05, compared to MCSFA group

^d^*p* < 0.05, compared to MUFA group

^e^*p* < 0.05, compared to n-3 PUFA group

^f^*p* < 0.05, compared to n-6 PUFA group

**Fig. 4:**
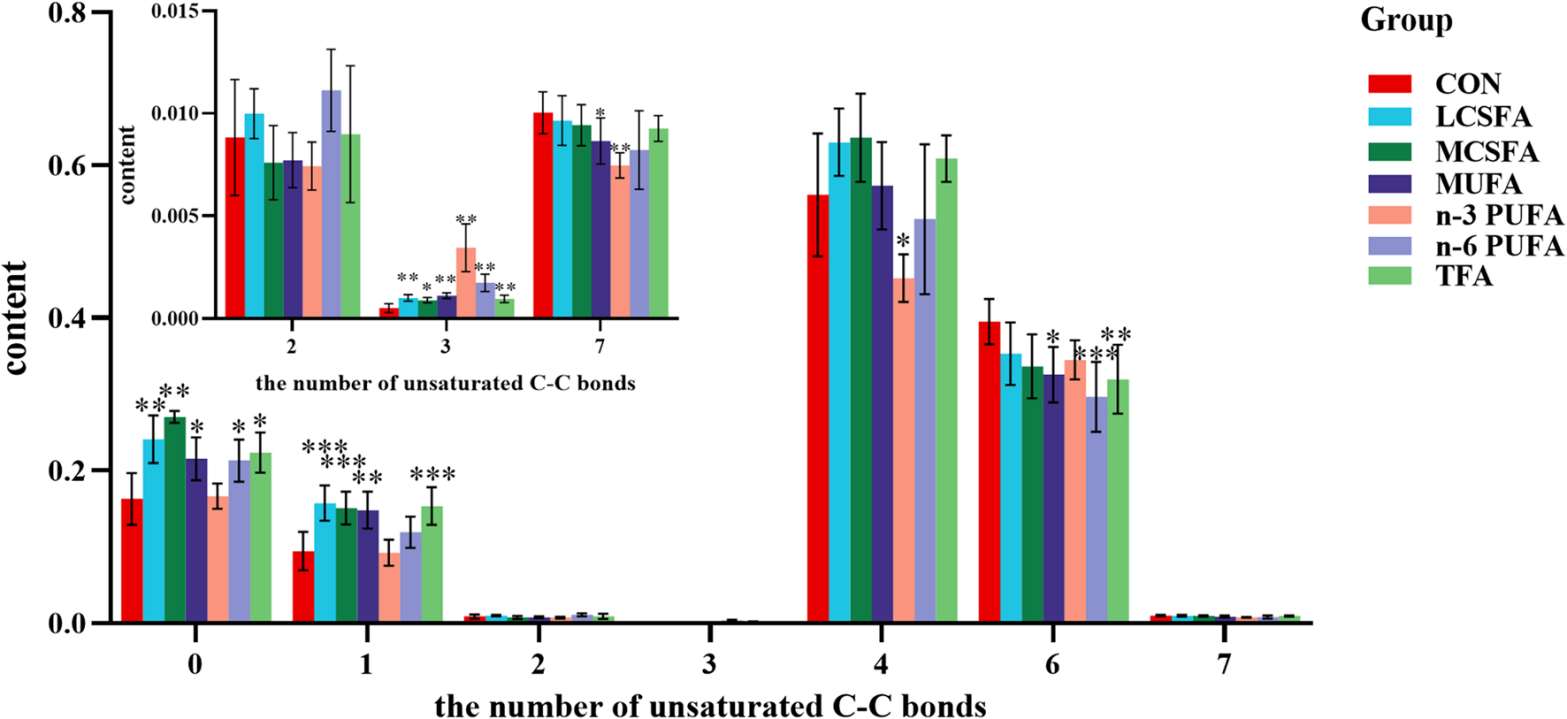
Unsaturation degree of total FA in brain of mice fed with HFD. *, **, ***denotes a statistically significant difference (*p* < 0.05, 0.01, 0.001) compared to the CON group, the lipid contents were indicated in the figure in % (Mean ± SD, n = 6)

Overall, the intake of different kinds of dietary fatty acids had a great effect on the saturation of FA in the brain. Majority of lipids were polyunsaturated with either 4 or 6 double bonds. Lipids with the 4 unsaturated bonds were the most abundant, and fully saturated lipids were the third. Compared with the CON group, the increase in the intake of fatty acids elevated the saturation of brain lipids and the proportion of lipids with low unsaturation. Among them, the lipids saturation of MUFA group showed an upward trend: the contents of lipids with high unsaturation decreased, the proportion of lipids containing 0, 1 and 3 unsaturated bond increased (*p* < 0.05), the lipids containing 6 to 7 unsaturated bonds decreased (*p* < 0.05), and the highest proportion of unsaturated lipids was the one containing 4 double bonds. As whole, the saturation and the proportion of saturated lipids (*p* < 0.05) increased in MCSFA group (Fig. 4).

**Fig. 5:**
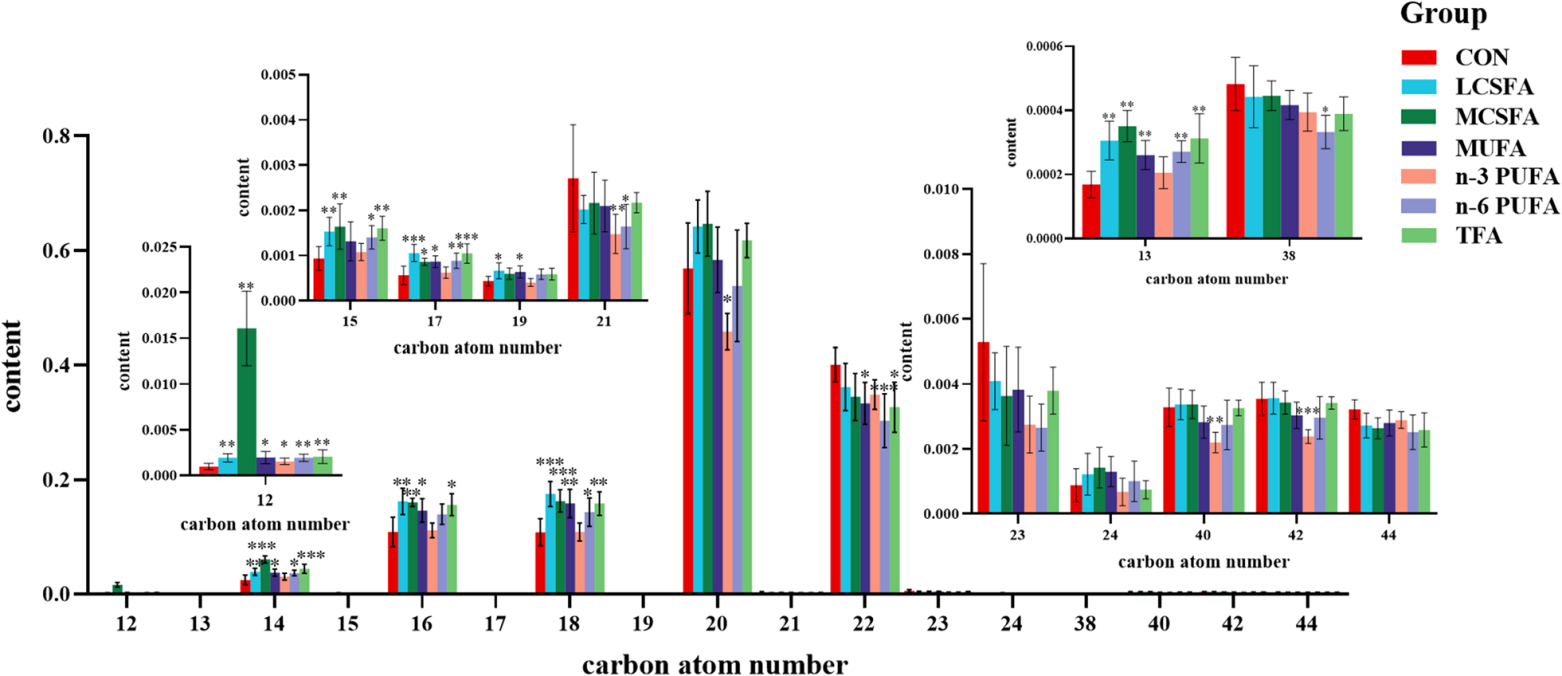
The carbon atom numbers of FA in brain of mice fed with HFD. *, **, ***denotes a statistically significant difference (*p* < 0.05, 0.01, 0.001) compared to the CON group, the lipid contents were indicated in the figure in % (Mean ± SD, n = 6)

Corresponding to the number of double bonds, majority of FA found in the brain of mice carry 16 or more total carbons and most of them were even 20 and 22 carbons, whereas < 13C and 23–44C length lipids were less abundant in all groups. The levels of lipid molecules with 12–19 carbon atoms showed a significantly higher trend in most of LCSFA, MCSFA, MUFA, n-6 PUFA and TFA groups compared to CON group (*p* < 0.05) (Fig. 5), and this trend was no longer significant with the extension of the carbon chain.

### The variations in the abundance of sphingolipids (SP) in brain of HFD-fed mice

**Fig. 6:**
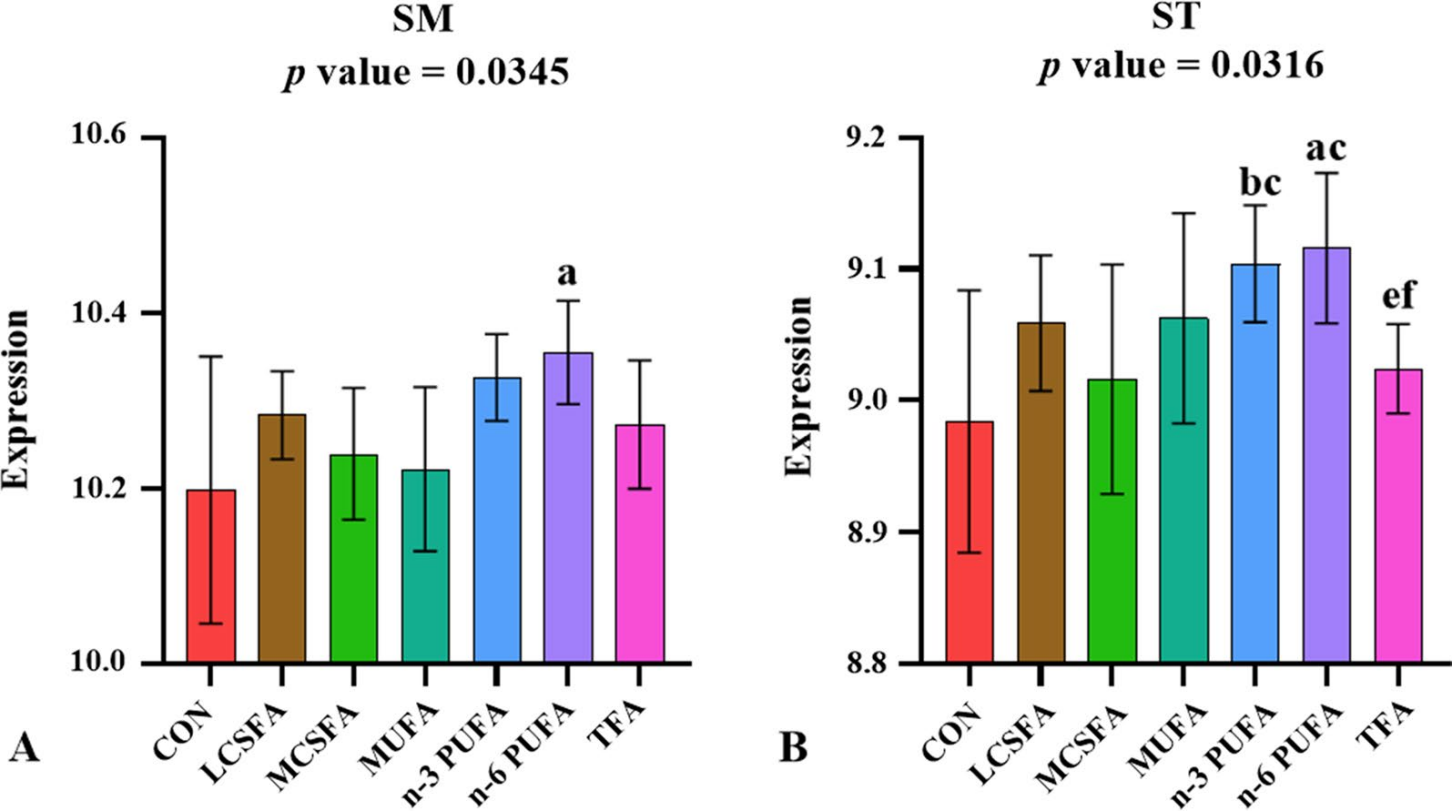
Relative abundance of the ST and SM class in SP main class in brain of mice fed with HFD. **a:** *p* < 0.05, compared to CON group; **b:** *p* < 0.05, compared to LCSFA group; **c:** *p* < 0.05, compared to MCSFA group; **d:** *p* < 0.05, compared to MUFA group; **e:** *p* < 0.05, compared to n-3 PUFA group; **f:** *p* < 0.05, compared to n-6 PUFA group. Mean ± SD, n = 6

The intake of n-6 PUFA increased the contents of sphingomyelin (SM) and ST in the brain of mice compared with CON group significantly (*p* < 0.05) (Fig. 6). This was consistent with the change trend of SP content in mice with FMT.

### The variations in the abundance of GP in hippocampus of mice with FMT

**Fig. 7:**
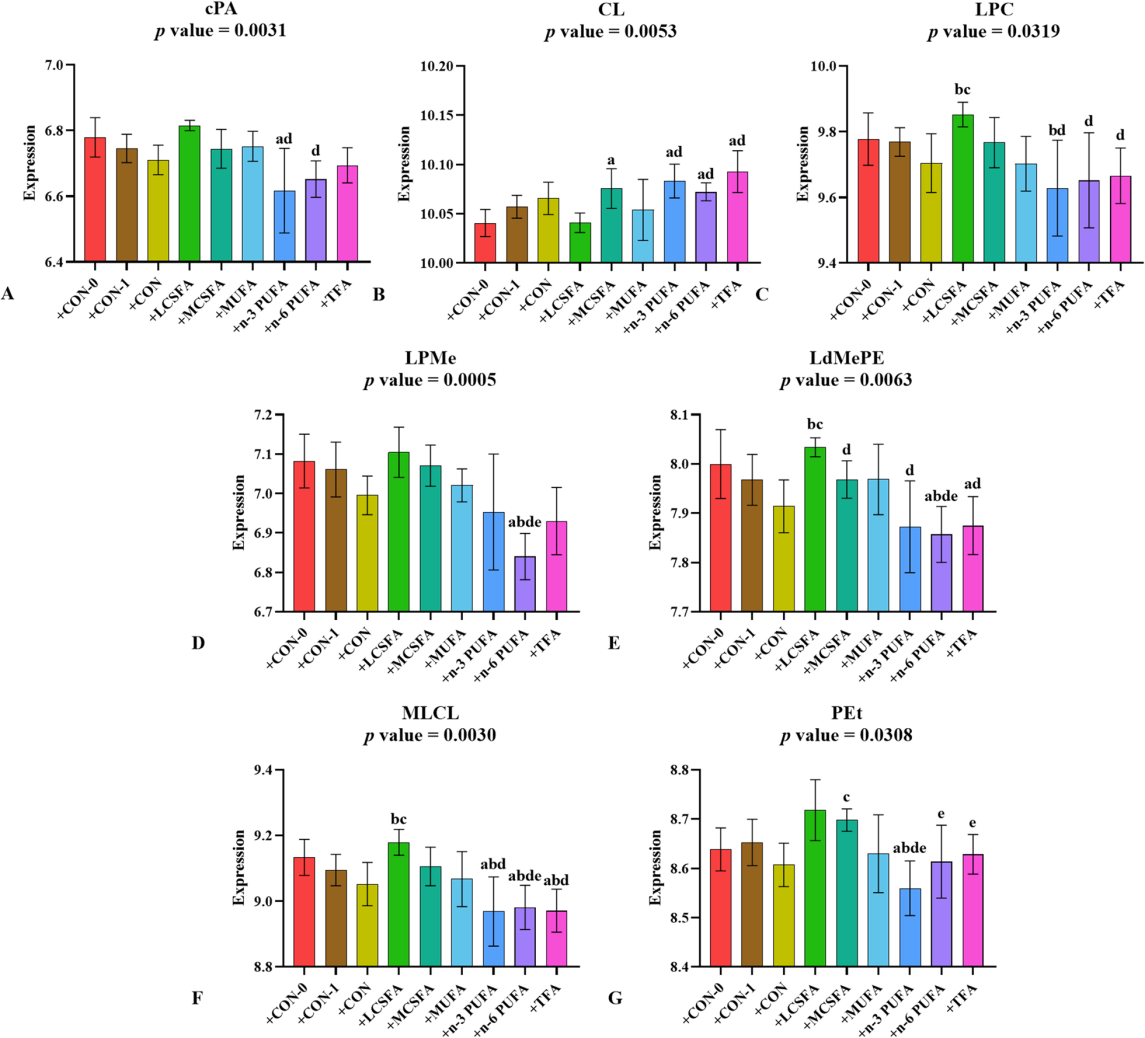
Relative abundance of GP in brain of mice with FMT. **A**–**G:** GP as a main class can be subdivided into classes: cPA, CL, LPC, LPMe, LdMePE, MLCL and PEt. **a:** *p* < 0.05, compared to + CON-0 group; **b:** *p* < 0.05, compared to + CON-1 group; **c:** *p* < 0.05, compared to + CON group; **d:** *p* < 0.05, compared to + LCSFA group; **e:** *p* < 0.05, compared to + MCSFA group; **f:** *p* < 0.05, compared to + MUFA group; **g:** *p* < 0.05, compared to + n-3 PUFA group; **h:** *p* < 0.05, compared to + n-6 PUFA group. Mean ± SD, n = 3–5

Dietary fatty acid intake of fecal donor mice was essential for lipid types and contents in hippocampal tissue of mice following FMT. Compared with + CON-1 and + CON groups the contents of lysophosphatidylcholine (LPC), lysodi-methylphosphatidylethanolamine (LdMePE), and monolysocardiolipin (MLCL) increased in + LCSFA group (*p* < 0.05) (Fig. 7C, E, F). Feces from mice fed with MCSFA also drove up the content of cardiolipin (CL) and PEt in the hippocampus of recipient mice (*p* < 0.05) (Fig. 7B, G). FMT from mice fed with n-3 PUFA, n-6 PUFA and TFA increased the content of CL compared with + CON-0 groups (*p* < 0.05) (Fig. 7B). When compared with + CON-0, + CON-1, + LSCFA and + MSCFA groups, the content of LPMe, LdMePE and MLCL decreased in the hippocampus of recipient mice after n-6 PUFA-fed FMT (*p* < 0.05) (Fig. 7D–F).

CL level in hippocampus of recipient mice was elevated in + n-3 PUFA group compared with + CON-0, + CON-1 or + LSCFA groups (*p* < 0.05) (Fig. 7B). In contrast, the FMT from n-3 PUFA diet mice reduced the contents of cPA, LPC, MLCL and PEt (*p* < 0.05) (Fig. 7A, C, F, G).

### The variations in the abundance of FA in hippocampus of mice with FMT

**Fig. 8:**
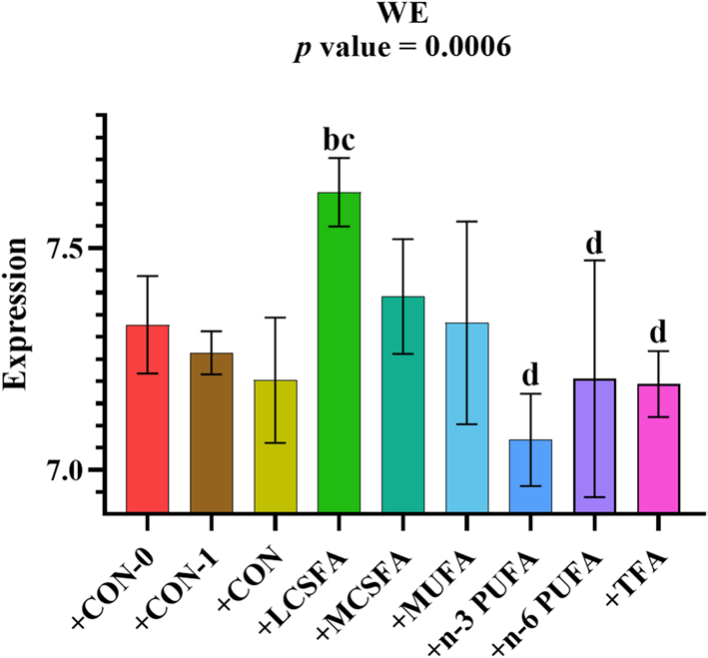
Relative abundance of WE class in FA main class in brain of mice with FMT. **a:** *p* < 0.05, compared to + CON-0 group; **b:** *p* < 0.05, compared to + CON-1 group; **c:** *p* < 0.05, compared to + CON group; **d:** *p* < 0.05, compared to + LCSFA group; **e:** *p* < 0.05, compared to + MCSFA group; **f:** *p* < 0.05, compared to + MUFA group; **g:** *p* < 0.05, compared to + n-3 PUFA group; **h:** *p* < 0.05, compared to + n-6 PUFA group. Mean ± SD, n = 3–5

An increase in wax ester (WE) content was observed at the + LCSFA group compared to + CON, + CON-1, + n-3 PUFA, + n-6 PUFA, and + TFA groups (*p* < 0.05) (Fig. 8).

### The variations in the abundance of SP in hippocampus of mice with FMT

**Fig. 9:**
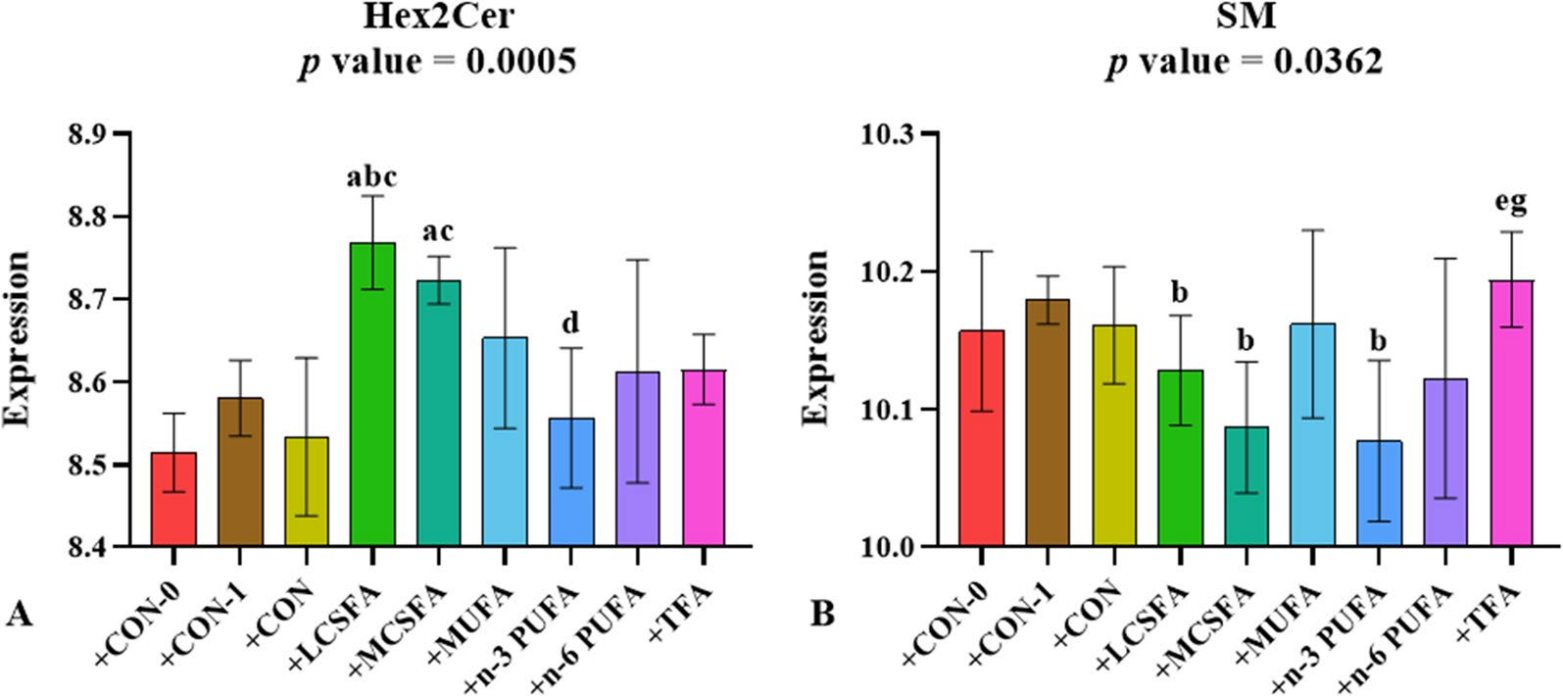
Relative abundance of SP in brain of mice with FMT. **A**, **B:** SP as a main class can be subdivided into classes: Hex2Cer and SM. **a:** *p* < 0.05, compared to + CON-0 group; **b:** *p* < 0.05, compared to + CON-1 group; **c:** *p* < 0.05, compared to + CON group; **d:** *p* < 0.05, compared to + LCSFA group; **e:** *p* < 0.05, compared to + MCSFA group; **f:** *p* < 0.05, compared to + MUFA group; **g:** *p* < 0.05, compared to + n-3 PUFA group; **h:** *p* < 0.05, compared to + n-6 PUFA group. Mean ± SD, n = 3–5

FMT from HFD feeding mice made recipient mice exhibit different degrees of increase in Hex2Cer content, but none reached significance in + MUFA, + n-6 PUFA and + TFA groups (*p* > 0.05) (Fig. 9A). Compared to the + CON-1 group, the amount of SM was reduced in + LCSFA, + MCSFA and + n-3 PUFA groups (*p* < 0.05) (Fig. 9B).

## Discussion

The brain lipidome determines the location and function of proteins on the neuronal cell membrane [24]. Lipids act as neurotransmitters or other signaling molecules [25]. The composition of brain lipids is influenced by certain factors, such as nutrition, environmental factor and behavioral activity [26].

Studies have shown that HFD could damage neurogenesis through lipid peroxidation and reduce brain-derived neurotrophic factor [27]. HFD has been also associated with reduced hippocampal volume, which regulates learning ability, memory and mood [28]. Although the effect of HFD on brain health has been studied in pathogenesis, the relationship between HFD and brain fat changes has not been widely studied. Higher lipid saturation may impair cell proliferation [29] and membrane fluidity [30]. Overall, our LCSFA, MCSFA, MUFA, n-6 PUFA, TFA supplements have elevated lipid saturation in brain. In addition, the increase of acyl chain lengths in brain were observed in AD patients and transgenic familial AD mice [18]. These findings were consistent with our results.

In mammals, phospholipase D (PLD)-mediated PA can resist apoptosis and promote mitophagy [31]. The increased expression and activity of PLD occur in many human cancer cells [32]. PLD also causes damage to cells by activating nicotinamide adenine dinucleotide phosphate to overproduce reactive oxygen species [33, 34]. It can be considered that PA has a pro-oncogenic role and cell oxidative damage effects. In addition, study also has shown that, elevated plasma PE levels are positively correlated with the incidence rate of type II diabetes (T2D) and metabolic syndrome [35]. ST might be involved in the regulation of inflammatory factors which decreased the activation of nuclear factor kappa-B translocation into the nucleus, ST hindered the localization of toll-like receptor 4 within lipid rafts, thereby diminishing the TLR4 signaling pathway, ST also had anti-inflammatory effects in the peripheral immune system [36]. ST downregulated systemic inflammation through a the Cluster of Differentiation 1D Glycoprotein dependent pathway mediated by type II Natural killer T cells [17]. However, it has also been shown that ST could induce pathological inflammatory responses in glial and brain-resident immune cells within the brain [37]. In our study, we found that HFD with n-6 PUFA increased the content of PE, PA and ST in the brain of mice, which indicated that excessive n-6 PUFA intake might exacerbate cell oxidative damage and metabolic impairment, but the effects on inflammatory response were inconclusive.

The high-fat diet might facilitate inflammatory responses, LPG might be mediated by formyl peptide receptor like-1 as cell responses regulator so that might show a potential anti-inflammatory effect [16]. A uniform decrease in LPG expression was observed in the experimental groups in our study, which might be a possible mechanism of HFD-fed intracerebral inflammation. The converse was seen with the AcCa, rising in its expression level caused by diet had a positive effect on the brain. It was consistent with our research: except for n-3 PUFA group, the content of AcCa in all HFD groups increased significantly compared with CON group (*p* < 0.05). That is, HFD could increase the content of AcCa in the brain regardless of the types of fatty acids intake. In the form of AcCa, carnitine carried activated long-chain fatty acids from cytoplasm into the mitochondria for subsequent oxidation and energy production, so it was essential for fatty acid oxidation and energy supply. Functionally, AcCa might alter the composition of cell membrane, stabilize cellular membrane, improve mitochondrial function and increase antioxidant activity in the brain [38]. The increase of AcCa content in the brain was associated with antidepressant effects. Carnitine also helped to remove the medium and short chain fatty acids which came from normal metabolic processes. And evidence derived from clinical populations indicated demonstrated elevations in plasma acylcarnitine concentrations in obese [39]. Since long-chain AcCa might inhibit or disrupt the intracellular stage of insulin signaling [40], patients with T2D and insulin resistance often appeared AcCa perturbations [41].

SM was abundant in the myelin sheath which also is an important SP in the brain [42]. By nuclear magnetic resonance, the study has been found an increased SM concentration in brain of patients diagnosed with AD. Targeted metabolomics analyses of brain tissue in AD cases showed that, the higher SM concentration, the more severe pathological changes, the higher abnormal cognition risk [43]. Accumulation of SM in brain impaired the enzymolysis of amyloid precursor protein C-terminal fragments, thus promoting the formation of characteristic amyloid beta peptide (Aβ) in AD [44]. Grimm *et al.* [45] have pointed out that Aβ42 could downregulate SM through sphingomyelinases activation. Both results thus mutually corroborated each other. Alternatively, SM levels seem to play an important role in regulating inflammation. SM was synthesized by sphingomyelin synthase (SMS), and sphingomyelin synthase 2 (SMS2) deficiency could reduce plasma membrane SM levels and attenuate nuclear factor kappa-B activation in macrophages and human embryonic kidney 293 cells [46]. Previous study has found a correlation between plasma SM and interleukin-6 [47]. Interestingly, the increase of SM (d18:1/24:0) or SM (d18:1/16:0) strongly induced intercellular adhesion molecule 1 and inducible nitric oxide synthase expression in macrophages, suggesting that SM was regulating macrophage activation and inflammation [48]. In our research, result suggested that high n-6 PUFA intake could increase SM expression which might be harmful to cognitive function with pro-inflammatory effect.

Foreign and domestic studies have shown that gut microbiota can be involved in the bidirectional regulation of the gastrointestinal tract and central nervous system through four pathways: nerve, metabolism, neuroendocrine and immunity, that is, the "gut microbiota-gut-brain axis" [49, 50]. Chronic consumption of HFD increased the ratio of *Firmicutes* to *Bacteroidetes* in adult (25 to 45 years old) compared with other age groups [51]. Animal study has suggested that a certain dose of n-3 PUFA could regulate the diversity, and abundance of gut microbiota, increase beneficial *Mycoplasmataceae* and *Firmicutes* levels in the gut and decrease gram-positive *Clostridium* levels in Kunming mice [52]. Our previous finding has shown that rats feeding with 1% and 8% TFA for 8 weeks significantly induced obesity, and the abundance of *Bacteroides* increased as well as *Muribaculaceae* decreased [53]. It has been shown that the gut microbiota can be independently involved in some organismal responses and can transmit certain properties of the donor into the recipient host [54, 55]. FMT is the persuasive way to explore the functions and specific mechanisms of the gut microbiota in dietary factors and brain lipids metabolites.

CL oxidation is involved in aging and mitochondrial bioenergetics change contributing to brain mitochondrial dysfunction caused by aging. Cerebral aging correlated with the occurrence of senile neurodegenerative conditions [56]. It was reported that both quantity and quality of the dietary fatty acid, including CL [57], altered lipids compositions in the mitochondrial membrane. MLCL, the three-tailed variant of CL, was predominantly distributed in the mitochondria. CL was remodeled from MLCL by the enzyme tafazzin. Tafazzin mutations had an impact on the transition from MLCL to CL and affected the function of mitochondria which was known as Barth syndrome. Barth syndrome included symptoms of cognitive deficits and hippocampus might serve as a potential treatment target for this disease [58]. MLCL was not typically detected in healthy tissues [59], an increase in MLCL indicated the remodeling of CL in brain mitochondrial was subjected to interference. In our study, LCSFA-fed fecal microbiota decreased CL and increased MLCL, in contrast, PUFA-fed fecal microbiota decreased MLCL and increased CL. Fecal microbiota fed with high SFA intake might alter energy metabolism and mitochondrial functional status, and might even inhibit the transition from MLCL to CL and thus impair cognition.

The digestion of WE released unsaturated fatty acids in the colon and activated G-protein-coupled receptor 120 in immune cells, which secreted hormones that controlled sugar and fat metabolism [60], reduced fat deposition in the liver and abdomen, and provided increased insulin sensitivity [61]. Supplementation of WE had significant anti-obesity effect, reduced obesity-related inflammation, and improved glucose tolerance and aerobic capacity [62]. WE as dominant energy storage lipid has been found in nervous system of other mammals [15]. In the meanwhile, as an endogenous inflammation-regulatory phospholipid, LPC has been associated with immunomodulatory functions of central nervous system glia. Saturated (LPC (16:0) and LPC (18:0)) and monounsaturated (LPC (18:1)) LPC had certain pro-inflammatory effects, such as the expression of adhesion molecules, the release of chemokines and the increase of reactive oxygen species production [63]. Unsaturated LPC (20:4) and LPC (22:6) exerted anti-inflammatory property by counteracting LPC (16:0)-induced inflammation and promoting formation of anti-inflammatory factors including interleukin-4 and interleukin-10 [64]. Our results showed that LCSFA-fed fecal microbiota up-regulated the expression of anti-obesity related WE and inflammation related LPC in mice brain.

Moreover, there was a strong positive correlation between inflammatory markers and hexanoylceramide (HexCer) species containing C16:0, C20:0 and C24:1 fatty acids [65]. There were strong correlations between adenosine triphosphate binding cassette transporter A7 genotype which was associated with an increased risk of AD and Hex2Cer [66]. Hex2Cer could induces insulin resistance [67]. Compared with normal mice, the content of Hex2Cer in the liver of obese T2D mice was increased [68]. Meanwhile, among overweight and obese populations, Hex2Cer was in positive correlation with the interleukin-10 [69]. In our findings, fact that the intake of a high SFA diet was associated with increase of Hex2Cer. This result was also confirmed in human study [70].

The gut microbiota comprises a large and diverse community that plays an important regulatory role in host physiological metabolism [71]. Dietary composition was known to alter the composition of the gut microbiome which in turn modified the local or systemic effects produced by the microbial community on the host [72]. Both SFA and TFA could lead to endothelial dysfunction, contribute to gut barrier injury, reduce the expression of tight junction proteins, then cause inflammatory cell infiltrates and eventually induce imbalances in gut microbiota [73, 74]. In addition, gut microbiota is the key to the mechanism of diet-induced cognitive impairment [49]. Wu *et al.* [75] found decline and disorganization of neurons in hippocampus of diet-fed mice accompanied by varying degrees of damage [75]. In our study, FMT was used to elucidate the effect of different types of dietary fatty acids mediated by gut microbiota on brain lipids metabolism of recipient mice. There were significant differences in the composition and structure of brain tissue in + LCSFA, + MCSFA, + MUFA, + n-3 PUFA, + n-6 PUFA and + TFA groups of mice with FMT. Although the lipid metabolic characteristics of donors were not completely replicated in recipients through gut microbiota fed with different types of dietary fatty acids, the disordered flora still had a negative effect on the brain tissue composition and central nervous system function of recipient mice. Our results suggest that gut microbiota and its metabolites played a vital role in brain lipidomics, providing new evidence and ideas for the specific pathway of the "gut microbiota-gut-brain axis".

## Conclusions

The study dealt with the impact of different types of fatty acids on lipid composition and lipid proportion of brain. Our results suggested that dietary n-3 PUFA, n-6 PUFA and LCSFA have a greater effect on brain lipids such as LPG and AcCa. Dietary fatty acids-fed gut microbiota could also have effects on lipid composition in the brain. SFA-fed gut microbiota up-regulated LPC, WE, and Hex2Cer levels in the brain. SFA-fed and PUFA-fed gut microbiota could regulate the interconversion between MLCL and CL. The content changes of these lipids might further affect the physiological functions they are involved in.

## Supplementary Information

**Additional file 1**: **Table S1** PE expression in the brain of mice fed with different dietary fatty acids (Mean ± SD, n=6). *, **, ***denotes a statistically significant difference (*p* < 0.05, 0.01, 0.001) in all groups. a: *p* < 0.05, compared to CON group; b: *p* < 0.05, compared to LCSFA group; c: *p* < 0.05, compared to MCSFA group; d: *p* < 0.05, compared to MUFA group; e: *p* < 0.05, compared to n-3 PUFA group; f: *p* < 0.05, compared to n-6 PUFA group.

## Data Availability

The datasets used during the current study are available from the corresponding author upon reasonable request. The data that support the findings of this study are available from Shanghai Majorbio Bio-Pharm Technology Co., Ltd. (https://www.majorbio.com/) but restrictions apply to the availability of these data, which were used under license for the current study, and so are not publicly available. Data are however available from the authors upon reasonable request and with permission of Shanghai Majorbio Bio-Pharm Technology Co., Ltd. (https://www.majorbio.com/).

## Abbreviations

AA: Arachidonic acid
AcCa: Acyl-carnitines
AD: Alzheimer's disease
ANOVA: Analysis of variance
Aβ: Amyloid beta peptide
BiotinylPE: Biotinyl-phosphatidylethanolamine
Cer: Ceramide
CL: Cardiolipin
CON: Control
DHA: Docosahexaenoic acid
DLCL: Dilyso-cardiolipin
dMePE: Dimethylphosphatidylethanolamine
DPA: Docosapentaenoic acid
dPE: Diacyl-phosphatidylethanolamine
EPA: Eicosapentaenoic acid
FA: Fatty acyl
FMT: Fecal microbiota transplant
GP: Glycerol phospholipid
Hex2Cer: Dihexosylceramide
HexCer: Hexanoylceramide
HFD: High-fat diet
LC-MS: Liquid chromatography-mass spectrometry
LCSFA: Long-chain saturated fatty acid
LdMePE: Lysodi-methylphosphatidylethanolamine
LPC: Lysophosphatidylcholine
LPE: Lysophosphatidylethanolamine
LPG: Lysophosphatidylgylcerol
MCSFA: Medium-chain saturated fatty acid
MePC: Methylphosphocholine
MLCL: Monolysocardiolipin
MUFA: Monounsaturated fatty acid
n-3 PUFA: N-3 polyunsaturated fatty acid
n-6 PUFA: N-6 polyunsaturated fatty acid
PA: Phosphatidic acids
PC: Phosphatidylcholine
PE: Phosphatidylethanolamine
PE-pl: Alkenylacyl-phosphatidylethanolamine or phosphatidylethanolamine plasmalogen
PI: Phosphatidylinositol
PLD: Phospholipase D
PS: Phosphatidylserine
SD: Standard deviation
SFA: Saturated fatty acid
SM: Sphingomyelin
SMS: Sphingomyelin synthase
SP: Sphingolipid
SPF: Specific pathogen free
ST: Sulfatide
T2D: Type II diabetes
TFA: Trans fatty acid
WE: Wax ester
