# Supplementary material for: Lipidomic analysis of brain and hippocampus from mice fed with high-fat diet and treated with fecal microbiota transplantation
Source: Nutr Metab (Lond). 2023 Feb 15;20:12. doi: 10.1186/s12986-023-00730-7 (PMC9930259; doi:10.1186/s12986-023-00730-7)
Supplement: Supplementary file 1 — Additional file 1: Table S1 PE expression in the brain of mice fed with different dietary fatty acids (Mean ± SD, n=6). *, **, ***denotes a statistically significant difference (p < 0.05, 0.01, 0.001) in all groups. a: p < 0.05, compared to CON group; b: p < 0.05, compared to LCSFA group; c: p < 0.05, compared to MCSFA group; d: p < 0.05, compared to MUFA group; e: p < 0.05, compared to n-3 PUFA group; f: p < 0.05, compared to n-6 PUFA group. [file 12986_2023_730_MOESM1_ESM.docx]

**Supplement table 1** PE expression in the brain of mice fed with different dietary fatty acids (Mean ± SD, n=6)

| **Expression** | **CON** | **LCSFA** | **MCSFA** | **MUFA** | **n-3 PUFA** | **n-6 PUFA** | **TFA** | ***p* value** |
| --- | --- | --- | --- | --- | --- | --- | --- | --- |
| PE (8:0e/10:0) | 7.867±0.179 | 7.858±0.114 | 7.843±0.151 | 7.856±0.114 | 7.919±0.123 | 7.938±0.145 | 7.791±0.096 | 0.583 |
| PE (8:1e/10:0) | 6.976±0.077 | 6.921±0.073 | 6.946±0.048 | 6.915±0.092 | 7.010±0.060 | 6.993±0.074 | 6.872±0.082 | 0.069 |
| PE (8:0/11:1) | 7.440±0.163 | 7.403±0.030 | 7.342±0.082 | 7.455±0.098 | 7.441±0.068 | 7.472±0.175 | 7.367±0.074 | 0.360 |
| PE (8:1e/12:0) | 7.589±0.204 | 7.643±0.074 | 7.633±0.090 | 7.603±0.056 | 7.478±0.091 | 7.637±0.120 | 7.601±0.074 | 0.135 |
| PE (8:0e/12:3) | 8.653±0.147 | 8.559±0.078 | 8.545±0.063 | 8.594±0.047 | 8.632±0.075 | 8.662±0.147 | 8.525±0.097 | 0.146 |
| PE (8:0e/12:4) | 7.161±0.143 | 7.114±0.082 | 7.132±0.039 | 7.106±0.052 | 7.077±0.062 | 7.167±0.148 | 7.126±0.047 | 0.658 |
| PE (4:0/17:1) | 7.166±0.158 | 7.156±0.088 | 7.098±0.033 | 7.150±0.108 | 6.945±0.067^abcd^ | 7.269±0.154^ce^ | 7.173±0.071^e^ | 0.004^**^ |
| PE (10:0/11:3) | 7.517±0.198 | 7.446±0.086 | 7.379±0.035 | 7.503±0.127 | 7.500±0.097 | 7.582±0.139 | 7.481±0.074 | 0.150 |
| PE (10:1e/12:3) | 7.599±0.203 | 7.643±0.074 | 7.633±0.090 | 7.603±0.056 | 7.478±0.091 | 7.637±0.120 | 7.601±0.074 | 0.132 |
| PE (10:0e/12:4) | 7.063±0.199 | 7.068±0.051 | 6.983±0.127 | 7.066±0.116 | 7.009±0.128 | 7.094±0.208 | 6.949±0.098 | 0.544 |
| PE (12:1e/10:4) | 6.330±0.201 | 5.823±0.143^a^ | 6.183±0.127^b^ | 5.535±0.141^abc^ | 5.026±0.294^abcd^ | 5.410±0.234^abce^ | 6.001±0.080^abcdef^ | <0.001^***^ |
| PE (11:0/12:4) | 7.166±0.158 | 7.156±0.088 | 7.098±0.033 | 7.150±0.108 | 6.945±0.067^abcd^ | 7.269±0.154^ce^ | 7.173±0.071^e^ | 0.004^**^ |
| PE (6:0/22:5) | 6.491±0.239 | 6.462±0.148 | 6.547±0.118 | 6.537±0.139 | 6.566±0.219 | 6.518±0.104 | 6.637±0.140 | 0.654 |
| PE (9:0/22:6) | 6.435±0.198 | 6.384±0.170 | 6.488±0.119 | 6.519±0.144 | 6.567±0.147 | 6.472±0.138 | 6.595±0.140 | 0.420 |
| PE (16:0/16:0) | 7.727±0.151 | 7.690±0.137 | 7.868±0.175 | 7.572±0.148^c^ | 7.980±0.177^abd^ | 7.965±0.149^abd^ | 7.390±0.070^abcef^ | <0.001^***^ |
| PE (16:0/16:1) | 7.883±0.034 | 7.772±0.014^a^ | 7.916±0.054^b^ | 7.785±0.046^ac^ | 7.916±0.095^bd^ | 7.765±0.047^ace^ | 7.761±0.033^ace^ | <0.001^***^ |
| PE (16:0p/16:1) | 7.712±0.079 | 7.703±0.038 | 7.909±0.054^ab^ | 7.742±0.041^c^ | 7.840±0.053^ab^ | 7.765±0.093^c^ | 7.707±0.055^ce^ | <0.001^***^ |
| PE (18:0/16:0) | 7.317±0.098 | 7.286±0.050 | 7.323±0.066 | 7.228±0.028 | 7.261±0.070 | 7.407±0.071^de^ | 7.278±0.081^f^ | 0.003^**^ |
| PE (16:0e/18:1) | 8.060±0.138 | 8.102±0.197 | 8.050±0.187 | 8.193±0.096 | 8.127±0.168 | 8.105±0.182 | 8.245±0.085 | 0.319 |
| PE (16:0p/18:1) | 9.274±0.032 | 9.267±0.058 | 9.288±0.036 | 9.312±0.028 | 9.359±0.073^b^ | 9.344±0.067 | 9.251±0.043^ef^ | 0.005^**^ |
| PE (16:1/18:1) | 8.293±0.074 | 8.190±0.075 | 8.306±0.112 | 8.023±0.091^abc^ | 8.020±0.089^abc^ | 8.150±0.096 | 8.150±0.060^c^ | <0.001^***^ |
| PE (16:0/18:2) | 8.412±0.261 | 8.436±0.269 | 8.236±0.206 | 8.583±0.036^c^ | 9.220±0.045^abcd^ | 8.613±0.050^bce^ | 8.632±0.020^bcde^ | <0.001^***^ |
| PE (16:0e/18:2) | 7.482±0.154 | 7.669±0.117 | 7.596±0.094 | 7.630±0.145 | 7.767±0.115^a^ | 7.670±0.133 | 7.614±0.069 | 0.016^*^ |
| PE (18:1p/16:1) | 8.233±0.084 | 8.201±0.028 | 8.237±0.062 | 8.215±0.044 | 8.342±0.044^abcd^ | 8.226±0.076^e^ | 8.169±0.050^e^ | 0.012^*^ |
| PE (16:0/18:3) | 7.484±0.090 | 7.424±0.076 | 7.643±0.114^b^ | 7.379±0.091^c^ | 7.554±0.094^d^ | 7.459±0.117^c^ | 7.473±0.064^cd^ | 0.007^**^ |
| PE (16:1/18:2) | 7.671±0.184 | 7.695±0.124 | 7.847±0.182 | 7.568±0.144 | 8.038±0.035^abd^ | 8.024±0.059^abd^ | 7.338±0.151^abcef^ | <0.001^***^ |
| PE (16:2e/18:1) | 6.488±0.229 | 6.624±0.265 | 6.640±0.205 | 6.750±0.153 | 6.525±0.395 | 6.573±0.127 | 6.617±0.280 | 0.660 |
| PE (14:0/20:4) | 6.783±0.104 | 6.792±0.102 | 7.110±0.098^ab^ | 6.764±0.107^c^ | 6.822±0.068^c^ | 6.840±0.103^c^ | 6.797±0.044^c^ | <0.001^***^ |
| PE (17:0/18:1) | 7.850±0.071 | 7.828±0.076 | 7.860±0.098 | 7.811±0.035 | 7.801±0.048 | 7.913±0.077 | 7.841±0.031 | 0.151 |
| PE (16:0p/19:1) | 8.183±0.148 | 8.162±0.058 | 8.163±0.079 | 8.116±0.110 | 8.256±0.073 | 8.146±0.285 | 8.108±0.058 | 0.224 |
| PE (15:0/20:3) | 7.864±0.142 | 7.873±0.141 | 7.853±0.126 | 7.833±0.088 | 7.759±0.119 | 7.899±0.104 | 7.859±0.049 | 0.460 |
| PE (20:4e/15:0) | 9.100±0.047 | 9.048±0.073 | 9.072±0.060 | 8.998±0.142 | 9.125±0.072 | 9.096±0.094 | 8.958±0.137 | 0.050^*^ |
| PE (18:0/18:0) | 8.232±0.078 | 8.227±0.049 | 8.209±0.044 | 8.339±0.070^c^ | 8.665±0.085^abcd^ | 8.456±0.036^abcde^ | 8.275±0.061^ef^ | <0.001^***^ |
| PE (18:0/18:1) | 7.101±0.622 | 7.352±0.416 | 7.102±0.438 | 7.094±0.286 | 6.911±0.197 | 7.173±0.488 | 7.235±0.325 | 0.591 |
| PE (18:0p/18:1) | 9.594±0.063 | 9.621±0.052 | 9.606±0.065 | 9.663±0.043 | 9.696±0.067^a^ | 9.686±0.066^a^ | 9.585±0.035^def^ | 0.015^*^ |
| PE (18:1/18:1) | 9.653±0.035 | 9.627±0.045 | 9.635±0.052 | 9.677±0.059 | 9.454±0.052^abcd^ | 9.682±0.031^e^ | 9.574±0.033^acdef^ | <0.001^***^ |
| PE (18:1e/18:1) | 8.649±0.034 | 8.622±0.056 | 8.629±0.047 | 8.679±0.019^c^ | 8.729±0.045^abc^ | 8.715±0.047^abc^ | 8.628±0.031^def^ | 0.001^**^ |
| PE (18:1p/18:1) | 9.823±0.050 | 9.791±0.046 | 9.783±0.073 | 9.831±0.035 | 9.849±0.054 | 9.829±0.064 | 9.743±0.055 | 0.063 |
| PE (18:1/18:2) | 8.793±0.246 | 8.893±0.133 | 8.671±0.587 | 8.969±0.058 | 9.479±0.055^abcd^ | 9.277±0.056^abcde^ | 9.196±0.041^abcdef^ | <0.001^***^ |
| PE (18:2p/18:1) | 7.390±0.269 | 7.741±0.180 | 7.624±0.191 | 7.647±0.151 | 7.548±0.316 | 7.725±0.096 | 7.574±0.179 | 0.100 |
| PE (18:2/18:2) | 5.545±0.488 | 5.755±0.257 | 5.645±0.317 | 6.043±0.176^a^ | 7.959±0.043^abcd^ | 6.922±0.044^abcde^ | 5.994±0.268^ef^ | <0.001^***^ |
| PE (16:0/20:4) | 8.418±0.177 | 8.425±0.095 | 8.463±0.117 | 8.519±0.083^abcd^ | 8.187±0.175^e^ | 8.476±0.110^e^ | 8.584±0.060 | <0.001^***^ |
| PE (16:0e/20:4) | 7.439±0.119 | 7.372±0.124 | 7.468±0.159 | 7.411±0.106 | 7.405±0.160 | 7.464±0.084 | 7.646±0.088^abcdef^ | 0.033^*^ |
| PE (16:0p/20:4) | 9.310±0.104 | 9.288±0.104 | 9.310±0.095 | 9.304±0.097 | 9.270±0.108 | 9.365±0.101 | 9.363±0.052 | 0.550 |
| PE (18:3/18:2) | 4.628±0.177 | 4.954±0.398 | 4.509±0.528 | 4.894±0.447 | 7.331±0.238^abcd^ | 5.891±0.122^abcde^ | 4.779±0.315^ef^ | <0.001^***^ |
| PE (18:4/18:1) | 6.494±0.077 | 6.495±0.101 | 6.546±0.212 | 6.510±0.115 | 6.641±0.150 | 6.618±0.182 | 7.081±0.051^abcdef^ | 0.004^**^ |
| PE (16:1/20:4) | 8.293±0.074 | 8.190±0.075 | 8.306±0.112 | 8.024±0.090^abc^ | 8.020±0.089^abc^ | 8.150±0.096 | 8.150±0.060^c^ | <0.001^***^ |
| PE (16:1p/20:4) | 7.326±0.074 | 7.290±0.124 | 7.586±0.146^ab^ | 7.298±0.084^c^ | 7.521±0.072^abd^ | 7.414±0.076^c^ | 7.420±0.050 | <0.001^***^ |
| PE (16:0p/20:5) | 6.508±0.224 | 6.339±0.220 | 6.629±0.208 | 6.856±0.196^ab^ | 8.157±0.054^abcd^ | 6.794±0.233^be^ | 7.009±0.134^abce^ | <0.001^***^ |
| PE (14:0/22:6) | 7.456±0.085 | 7.401±0.076 | 7.626±0.114^ab^ | 7.371±0.103^c^ | 7.546±0.092^d^ | 7.453±0.114^c^ | 7.469±0.060 | <0.001^***^ |
| PE (16:1e/20:5) | 4.907±0.389 | 4.470±0.400^a^ | 4.911±0.481 | 4.586±0.298 | 6.885±0.130^abcd^ | 4.603±0.212^e^ | 5.183±0.152^bdef^ | <0.001^***^ |
| PE (14:0p/22:6) | 7.244±0.329 | 7.123±0.098 | 7.397±0.181^b^ | 7.201±0.079^c^ | 7.053±0.088^cd^ | 7.387±0.059^bde^ | 7.233±0.085^bef^ | <0.001^***^ |
| PE (17:0/20:3) | 8.214±0.068 | 8.217±0.066 | 8.221±0.078 | 8.237±0.062 | 8.166±0.066 | 8.322±0.061^e^ | 8.205±0.047^f^ | 0.011^*^ |
| PE (17:1/20:3) | 8.069±0.079 | 8.082±0.063 | 8.031±0.090 | 8.053±0.070 | 8.002±0.096 | 8.082±0.083 | 8.109±0.049 | 0.348 |
| PE (17:0/20:4) | 7.850±0.071 | 7.828±0.076 | 7.860±0.098 | 7.811±0.035 | 7.801±0.048 | 7.913±0.077 | 7.841±0.031 | 0.151 |
| PE (15:0/22:6) | 7.862±0.131 | 7.869±0.138 | 7.852±0.126 | 7.826±0.092 | 7.765±0.118 | 7.903±0.108 | 7.863±0.048 | 0.472 |
| PE (18:0/20:1) | 9.032±0.120 | 9.004±0.117 | 9.024±0.181 | 8.987±0.089 | 8.896±0.076 | 9.196±0.100^e^ | 9.070±0.174 | 0.017^*^ |
| PE (18:0p/20:1) | 9.190±0.125 | 9.267±0.086 | 9.272±0.101 | 9.310±0.086 | 9.323±0.095 | 9.321±0.086 | 9.220±0.056 | 0.222 |
| PE (18:1e/20:1) | 9.638±0.067 | 9.667±0.042 | 9.659±0.051 | 9.663±0.042 | 9.499±0.079^abcd^ | 9.691±0.060^e^ | 9.610±0.076^e^ | <0.001^***^ |
| PE (20:0e/18:2) | 8.260±0.069 | 8.311±0.068 | 8.253±0.199 | 8.350±0.100 | 8.384±0.109 | 8.393±0.102 | 8.222±0.065 | 0.060 |
| PE (18:1p/20:1) | 9.611±0.084 | 9.627±0.152 | 9.582±0.125 | 9.622±0.097 | 9.645±0.140 | 9.715±0.069 | 9.539±0.074 | 0.077 |
| PE (18:1/20:2) | 8.393±0.084 | 8.367±0.081 | 8.367±0.123 | 8.329±0.100 | 8.425±0.054 | 8.351±0.096 | 8.277±0.108 | 0.216 |
| PE (18:0/20:3) | 9.821±0.056 | 9.801±0.062 | 9.786±0.058 | 9.830±0.042 | 9.896±0.072^c^ | 9.906±0.073^c^ | 9.807±0.043 | 0.005^**^ |
| PE (20:3e/18:0) | 8.592±0.048 | 8.565±0.050 | 8.588±0.057 | 8.623±0.014 | 8.536±0.186 | 8.641±0.046 | 8.572±0.038 | 0.115 |
| PE (18:1/20:3) | 8.198±0.068 | 8.156±0.121 | 8.202±0.137 | 8.137±0.070 | 8.367±0.038^abcd^ | 8.223±0.091 | 8.328±0.067^bd^ | <0.001^***^ |
| PE (16:0p/22:4) | 9.230±0.069 | 9.110±0.101 | 9.204±0.111 | 9.094±0.047 | 9.025±0.109^ac^ | 9.170±0.115 | 9.165±0.101 | 0.012^*^ |
| PE (18:0p/20:4) | 9.630±0.077 | 9.638±0.088 | 9.650±0.081 | 9.630±0.074 | 9.546±0.121 | 9.710±0.091 | 9.696±0.052 | 0.064 |
| PE (18:1/20:4) | 5.433±0.354 | 5.644±0.311 | 5.066±0.415^b^ | 5.805±0.545^c^ | 7.474±0.059^abcd^ | 6.232±0.243^abce^ | 5.508±0.194^ef^ | <0.001^***^ |
| PE (16:0/22:5) | 8.132±0.063 | 8.235±0.053 | 8.067±0.065^b^ | 8.119±0.080 | 8.431±0.117^abcd^ | 8.582±0.125^abcde^ | 8.316±0.048^acdf^ | <0.001^***^ |
| PE (18:1p/20:4) | 9.742±0.059 | 9.730±0.043 | 9.717±0.044 | 9.722±0.031 | 9.726±0.054 | 9.777±0.039 | 9.760±0.029 | 0.160 |
| PE (18:2/20:4) | 6.269±0.184 | 6.666±0.335 | 5.958±0.516^b^ | 6.662±0.282^ac^ | 8.420±0.073^abcd^ | 7.206±0.149^abcde^ | 6.625±0.249^acef^ | <0.001^***^ |
| PE (16:1/22:5) | 6.953±0.107 | 7.249±0.054^a^ | 6.923±0.069^b^ | 7.262±0.058^ac^ | 8.170±0.051^abcd^ | 7.833±0.102^abcde^ | 7.338±0.129^acef^ | <0.001^***^ |
| PE (16:0e/22:6) | 7.885±0.108 | 8.169±0.107^a^ | 7.894±0.095^b^ | 8.006±0.122 | 8.412±0.121^abcd^ | 8.574±0.146^abcd^ | 8.236±0.054^acdf^ | <0.001^***^ |
| PE (20:4e/18:2) | 8.232±0.129 | 8.369±0.048^a^ | 8.327±0.090 | 8.325±0.075 | 8.205±0.047^bcd^ | 8.452±0.058^abcde^ | 8.329±0.063^ef^ | <0.001^***^ |
| PE (18:2p/20:4) | 7.087±0.118 | 7.463±0.092^a^ | 7.004±0.170^b^ | 7.088±0.174^b^ | 7.361±0.065^acd^ | 7.891±0.063^abcde^ | 7.253±0.190^cf^ | <0.001^***^ |
| PE (18:1p/20:5) | 7.002±0.426 | 7.021±0.168 | 6.761±0.129^b^ | 7.496±0.431^ac^ | 8.512±0.071^abcd^ | 7.422±0.283^bce^ | 7.448±0.347^bce^ | <0.001^***^ |
| PE (16:0p/22:6) | 9.804±0.069 | 9.958±0.078 | 9.817±0.126 | 9.828±0.109 | 9.837±0.085 | 10.023±0.090^acde^ | 9.897±0.141 | 0.004^**^ |
| PE (16:1/22:6) | 4.700±0.215 | 5.017±0.150^a^ | 4.588±0.366^b^ | 5.115±0.206^ac^ | 6.873±0.045^abcd^ | 5.727±0.110^abcde^ | 4.854±0.132^def^ | <0.001^***^ |
| PE (18:3/20:4) | 5.667±0.137 | 5.755±0.257 | 5.601±0.331 | 6.043±0.176^ac^ | 7.959±0.043^abcd^ | 6.922±0.044^abcde^ | 5.875±0.272^ef^ | <0.001^***^ |
| PE (16:1e/22:6) | 4.414±0.323 | 4.376±0.504 | 4.231±0.325 | 4.695±0.426 | 7.559±0.082^abcd^ | 6.228±0.145^abcde^ | 4.844±0.260^acef^ | <0.001^***^ |
| PE (18:3e/20:4) | 9.737±0.084 | 9.958±0.078 | 9.788±0.156 | 9.773±0.129 | 9.781±0.122 | 10.023±0.090^acde^ | 9.874±0.176 | 0.002^**^ |
| PE (18:2p/20:5) | 5.943±0.230 | 6.256±0.315 | 6.045±0.255 | 6.437±0.267^ac^ | 7.574±0.058^abcd^ | 6.465±0.437^e^ | 6.380±0.303^ae^ | <0.001^***^ |
| PE (16:1p/22:6) | 8.086±0.129 | 8.025±0.112 | 8.041±0.116 | 8.025±0.101 | 8.099±0.086 | 8.131±0.097^b^ | 8.316±0.045^abcdef^ | 0.004^**^ |
| PE (18:4/20:5) | 3.941±0.038 | 3.944±0.042 | 3.964±0.054 | 3.955±0.022 | 6.292±0.127^abcd^ | 4.040±0.093^abde^ | 3.962±0.089^ef^ | <0.001^***^ |
| PE (20:4e/19:0) | 8.279±0.301 | 8.460±0.103 | 8.378±0.200 | 8.298±0.052 | 8.357±0.202 | 8.393±0.188 | 8.244±0.124 | 0.442 |
| PE (20:4e/19:1) | 8.513±0.087 | 8.516±0.088 | 8.526±0.100 | 8.546±0.059 | 8.503±0.114 | 8.536±0.104 | 8.468±0.051 | 0.811 |
| PE (17:0/22:6) | 8.194±0.071 | 8.202±0.058 | 8.209±0.076 | 8.226±0.060 | 8.152±0.150 | 8.306±0.062 | 8.197±0.046 | 0.091 |
| PE (17:1/22:6) | 8.074±0.079 | 8.090±0.059 | 8.050±0.092 | 8.058±0.068 | 8.002±0.090 | 8.092±0.081 | 8.124±0.047 | 0.180 |
| PE (18:0/22:1) | 8.081±0.050 | 8.040±0.074 | 8.089±0.061 | 8.061±0.053 | 7.970±0.056^ac^ | 8.132±0.074^e^ | 8.092±0.041^e^ | 0.002^**^ |
| PE (20:5/20:5) | 4.640±0.326 | 5.005±0.139^a^ | 4.671±0.356 | 5.208±0.085^abc^ | 6.883±0.044^abcd^ | 5.682±0.167^abcde^ | 4.940±0.129^def^ | <0.001^***^ |
| PE (18:0p/22:1) | 7.815±0.149 | 7.965±0.071 | 7.930±0.112 | 7.964±0.136 | 7.962±0.099 | 8.019±0.090^a^ | 7.878±0.057 | 0.048^*^ |
| PE (20:1/20:1) | 7.719±0.067 | 7.735±0.189 | 7.667±0.179 | 7.760±0.174 | 7.782±0.160 | 7.864±0.133 | 7.695±0.076 | 0.340 |
| PE (18:1p/22:1) | 8.739±0.081 | 8.791±0.062 | 8.748±0.151 | 8.813±0.060 | 8.768±0.101 | 8.822±0.080 | 8.740±0.068 | 0.525 |
| PE (18:0/22:3) | 8.013±0.118 | 7.639±0.069^a^ | 7.991±0.042^b^ | 7.873±0.077^b^ | 7.563±0.077^acd^ | 7.543±0.186^acd^ | 7.651±0.083^acd^ | <0.001^***^ |
| PE (20:3e/20:0) | 7.868±0.072 | 7.833±0.162 | 7.908±0.087 | 7.924±0.045 | 7.846±0.178 | 7.955±0.067 | 7.871±0.055 | 0.289 |
| PE (18:0p/22:3) | 8.339±0.068 | 8.019±0.101^a^ | 8.372±0.083^b^ | 8.230±0.044^bc^ | 7.921±0.073^acd^ | 7.876±0.050^abcd^ | 7.961±0.056^acd^ | <0.001^***^ |
| PE (20:1/20:3) | 7.489±0.146 | 7.408±0.110 | 7.414±0.103 | 7.432±0.089 | 7.512±0.092 | 7.505±0.165 | 7.385±0.057 | 0.308 |
| PE (18:0/22:4) | 9.032±0.120 | 9.004±0.117 | 9.024±0.181 | 8.987±0.089 | 8.896±0.076 | 9.196±0.100^e^ | 9.070±0.174 | 0.017^*^ |
| PE (18:1e/22:3) | 8.339±0.068 | 8.019±0.101^a^ | 8.372±0.083^b^ | 8.230±0.044^bc^ | 7.921±0.073^acd^ | 7.876±0.050^abcd^ | 7.961±0.056^acd^ | <0.001^***^ |
| PE (18:0p/22:4) | 9.584±0.068 | 9.612±0.038 | 9.604±0.052 | 9.637±0.041 | 9.561±0.046 | 9.686±0.045^ae^ | 9.632±0.023 | 0.002^**^ |
| PE (18:0/22:5) | 9.219±0.088 | 8.861±0.124^a^ | 9.128±0.146^b^ | 8.630±0.095^abc^ | 8.212±0.111^abcd^ | 8.513±0.109^abce^ | 8.994±0.059^adef^ | <0.001^***^ |
| PE (20:2e/20:3) | 7.668±0.073 | 7.695±0.138 | 7.678±0.084 | 7.686±0.018 | 7.703±0.075 | 7.707±0.041 | 7.672±0.054 | 0.624 |
| PE (18:0p/22:5) | 9.099±0.808 | 9.604±0.029 | 9.482±0.285 | 9.632±0.013^a^ | 8.572±0.430^bcd^ | 9.572±0.282^ae^ | 9.249±0.403^ef^ | <0.001^***^ |
| PE (20:3/20:3) | 8.704±0.092 | 8.670±0.068 | 8.660±0.056 | 8.773±0.064 | 8.959±0.049^abcd^ | 8.856±0.073^abc^ | 8.708±0.052^ef^ | <0.001^***^ |
| PE (18:0/22:6) | 7.266±0.078 | 7.120±0.077^a^ | 7.302±0.038^b^ | 7.142±0.098^ac^ | 6.856±0.360^acd^ | 7.062±0.119^ac^ | 6.958±0.297^ac^ | <0.001^***^ |
| PE (18:1p/22:5) | 8.775±0.072 | 8.759±0.049 | 8.730±0.051 | 8.846±0.055^bc^ | 8.851±0.050^abc^ | 8.854±0.050^bc^ | 8.806±0.049^c^ | 0.003^**^ |
| PE (18:0p/22:6) | 9.993±0.086 | 9.990±0.133 | 9.974±0.139 | 9.916±0.085 | 9.956±0.055 | 9.939±0.052 | 9.868±0.045 | 0.236 |
| PE (18:1/22:6) | 6.459±0.511 | 6.774±0.128 | 6.724±0.105 | 6.741±0.052 | 6.821±0.080 | 6.646±0.494 | 6.861±0.061 | 0.307 |
| PE (20:3/20:4) | 8.198±0.068 | 8.156±0.121 | 8.202±0.137 | 8.137±0.070 | 8.367±0.038^abcd^ | 8.223±0.091 | 8.328±0.067^bd^ | <0.001^***^ |
| PE (18:1e/22:6) | 6.648±0.218 | 6.770±0.189 | 6.693±0.144 | 6.891±0.100^c^ | 7.599±0.060^abcd^ | 6.776±0.551^e^ | 6.455±0.548^e^ | 0.001^**^ |
| PE (20:4/20:4) | 7.024±0.055 | 7.074±0.071 | 7.064±0.111 | 7.263±0.039^abc^ | 8.147±0.062^abcd^ | 7.280±0.117^abce^ | 7.146±0.049^ade^ | <0.001^***^ |
| PE (18:2/22:6) | 8.490±0.047 | 8.437±0.071 | 8.453±0.079 | 8.477±0.066 | 8.261±0.084^abcd^ | 8.630±0.087^abcde^ | 8.878±0.046^abcdef^ | <0.001^***^ |
| PE (18:2p/22:6) | 7.913±0.116 | 8.177±0.108^a^ | 7.922±0.079^b^ | 8.015±0.125 | 8.418±0.130^abcd^ | 8.576±0.137^abcd^ | 8.232±0.049^acdf^ | <0.001^***^ |
| PE (18:3/22:6) | 6.243±0.224 | 6.666±0.335^a^ | 5.959±0.517^b^ | 6.662±0.282^ac^ | 8.490±0.059^abcd^ | 7.206±0.149^abcde^ | 6.625±0.249^acef^ | <0.001^***^ |
| PE (18:3e/22:6) | 8.214±0.068 | 8.217±0.066 | 8.221±0.078 | 8.237±0.062 | 8.166±0.066 | 8.322±0.061^e^ | 8.205±0.047^f^ | 0.011^*^ |
| PE (18:1p/23:1) | 7.501±0.155 | 7.521±0.139 | 7.516±0.186 | 7.596±0.138 | 7.335±0.133 | 7.423±0.224 | 7.227±0.275 | 0.052 |
| PE (20:4e/21:1) | 7.705±0.067 | 7.714±0.233 | 7.640±0.189 | 7.751±0.175 | 7.782±0.160 | 7.864±0.133 | 7.695±0.076 | 0.256 |
| PE (20:4/22:6) | 6.602±0.186 | 6.547±0.135 | 6.544±0.199 | 6.694±0.135 | 6.736±0.072 | 6.631±0.144 | 6.526±0.133 | 0.135 |
| PE (20:5/22:6) | 7.013±0.058 | 7.074±0.080 | 7.057±0.110 | 7.263±0.040^abc^ | 8.146±0.063^abcd^ | 7.271±0.106^abce^ | 7.122±0.059^adef^ | <0.001^***^ |
| PE (18:1p/24:1) | 8.067±0.090 | 8.078±0.206 | 8.029±0.177 | 8.222±0.114 | 8.125±0.114 | 8.172±0.151 | 8.247±0.126 | 0.102 |
| PE (20:0/22:4) | 8.081±0.050 | 8.040±0.074 | 8.089±0.061 | 8.061±0.053 | 7.970±0.056^ac^ | 8.132±0.074^e^ | 8.092±0.041^e^ | 0.002^**^ |
| PE (20:0p/22:4) | 7.858±0.353 | 7.806±0.356 | 8.002±0.069 | 7.939±0.085 | 7.911±0.273 | 8.010±0.088 | 7.957±0.353 | 0.669 |
| PE (20:1e/22:4) | 8.269±0.098 | 8.250±0.115 | 8.296±0.127 | 8.235±0.031 | 7.873±0.270^abcd^ | 8.273±0.038^e^ | 8.186±0.245^e^ | 0.014^*^ |
| PE (20:0/22:6) | 6.880±0.136 | 6.824±0.140 | 6.841±0.168 | 6.851±0.279 | 7.149±0.109^bcd^ | 6.994±0.122 | 6.904±0.080 | 0.014^*^ |
| PE (20:3/22:4) | 9.093±0.036 | 9.071±0.055 | 9.145±0.129 | 9.093±0.050 | 8.859±0.064^abcd^ | 9.073±0.050^e^ | 9.113±0.052^e^ | 0.007^**^ |
| PE (20:1/22:6) | 7.489±0.146 | 7.408±0.110 | 7.414±0.103 | 7.432±0.089 | 7.512±0.092 | 7.505±0.165 | 7.385±0.057 | 0.308 |
| PE (20:2e/22:5) | 7.435±0.079 | 7.495±0.135 | 7.356±0.118 | 7.488±0.071 | 7.710±0.141^abcd^ | 7.596±0.119^c^ | 7.435±0.111^e^ | <0.001^***^ |
| PE (20:1p/22:6) | 7.574±0.046 | 7.561±0.039 | 7.603±0.082 | 7.646±0.027 | 7.665±0.063 | 7.731±0.124^abc^ | 7.622±0.047 | 0.002^**^ |
| PE (20:3/22:5) | 7.943±0.156 | 8.015±0.113 | 7.899±0.110 | 8.148±0.091^ac^ | 9.040±0.099^abcd^ | 8.246±0.090^abce^ | 7.931±0.026^def^ | <0.001^***^ |
| PE (20:2/22:6) | 8.158±0.126 | 7.711±0.105^a^ | 8.037±0.045^b^ | 7.470±0.112^abc^ | 7.090±0.325^abcd^ | 7.188±0.135^abcd^ | 7.812±0.050^acdef^ | <0.001^***^ |
| PE (20:3/22:6) | 9.029±0.169 | 8.909±0.097 | 8.867±0.122 | 9.003±0.070 | 9.095±0.110^bc^ | 8.996±0.110 | 8.862±0.023^adef^ | 0.004^**^ |
| PE (22:4/22:6) | 9.093±0.034 | 9.099±0.073 | 9.201±0.116 | 9.097±0.054 | 8.834±0.101^abcd^ | 9.084±0.052^e^ | 9.111±0.051^e^ | <0.001^***^ |
| PE (22:5/22:6) | 7.887±0.167 | 7.982±0.097 | 7.854±0.112 | 8.142±0.082^ac^ | 8.996±0.098^abcd^ | 8.224±0.088^abce^ | 7.922±0.035^def^ | <0.001^***^ |
| PE (22:6/22:6) | 8.985±0.182 | 8.932±0.107 | 8.869±0.125 | 9.005±0.056 | 9.056±0.121^c^ | 8.998±0.104 | 8.865±0.030^def^ | 0.023^*^ |
| PE (22:1/22:6) | 7.613±0.051 | 7.560±0.063 | 7.630±0.055 | 7.593±0.029 | 7.320±0.084^abcd^ | 7.617±0.029^e^ | 7.552±0.039^acef^ | <0.001^***^ |
| PE (22:2/22:6) | 6.803±0.135 | 6.613±0.045^a^ | 6.487±0.085^ab^ | 6.578±0.146^a^ | 6.581±0.098^a^ | 6.552±0.112^a^ | 6.591±0.064^a^ | 0.010^*^ |
| PE (38:1/10:4) | 7.868±0.067 | 7.790±0.062 | 7.808±0.095 | 7.835±0.163 | 7.863±0.101 | 7.856±0.085 | 7.884±0.065 | 0.618 |
| PE (31:0/24:0) | 6.259±0.100 | 6.224±0.075 | 6.260±0.067 | 6.270±0.077 | 6.332±0.059 | 6.296±0.101 | 6.285±0.047 | 0.352 |

*, **, ***denotes a statistically significant difference (*p* < 0.05, 0.01, 0.001) in all groups. a: *p* < 0.05, compared to CON group; b: *p* < 0.05, compared to LCSFA group; c: *p* < 0.05, compared to MCSFA group; d: *p* < 0.05, compared to MUFA group; e: *p* < 0.05, compared to n-3 PUFA group; f: *p* < 0.05, compared to n-6 PUFA group.
